# Energy transfer mechanism in ultrasonic impact and its single-cycle equivalent experimental methodology^☆^

**DOI:** 10.1016/j.ultsonch.2026.107910

**Published:** 2026-06-05

**Authors:** Qingshan Jiang, Tong Ran, Yongqing Lai, Peihan Lin, Pengbo Qian, Zhilong Xu, Fei Sun

**Affiliations:** a College of Marine Equipment and Mechanical Engineering, Jimei University, Xiamen 361000, China; b Engineering Research Center of Anti-Fatigue Manufacturing for Marine Equipment (Fujian Province), Xiamen 361000, China; c Department of Public Basic Courses, Nanjing University of Industry Technology, Nanjing 210000, China

**Keywords:** Ultrasonic impact treatment, Finite element simulation, Stress wave, Single-cycle, Equivalent impact treatment

## Abstract

Ultrasonic impact treatment is extensively employed to improve the surface quality and fatigue performance of engineered components. However, existing methodologies are limited by their inability to accurately characterize the transient behavior within a single impact cycle, which hinders the development of theoretically grounded parameter optimization. This paper investigates the stress wave propagation mechanism during single-cycle of ultrasonic impact and proposes an equivalent impact experimental analysis method for single-cycle ultrasonic impact treatment grounded in one-dimensional impact stress wave theory. The key innovation lies in decoupling the complex high-frequency process into a controllable single-cycle event based on a rigorously derived energy equivalence principle. An energy transfer model based on stress wave propagation was established, and the equivalence conditions for the impact parameters were subsequently derived. Complementary finite element models of ultrasonic impact treatment and equivalent impact treatment were constructed. Validation was performed via an equivalent impact test platform using 6061-T6 Aluminum Alloy and 45**#** steel. The stress wave history curve measured in the equivalent impact test closely replicates the theoretical ultrasonic impact curve, with errors in maximum amplitude and pulse width within 4.4% and 5.4% respectively. Comparison between simulation and equivalent impact test results reveals maximum depth and diameter errors below 3.7% and 7% respectively, with maximum residual stress error below 5.5% and hardness errors within 5.2% at different orientations. Electron Backscatter Diffraction analysis confirms highly consistent grain size distribution between the two techniques. This study lays a theoretical foundation for the visual investigation into the mechanism of ultrasonic impact and offers new insights for the exploration of various ultrasonic-based processes.

## Graphical abstract

**Figure:**
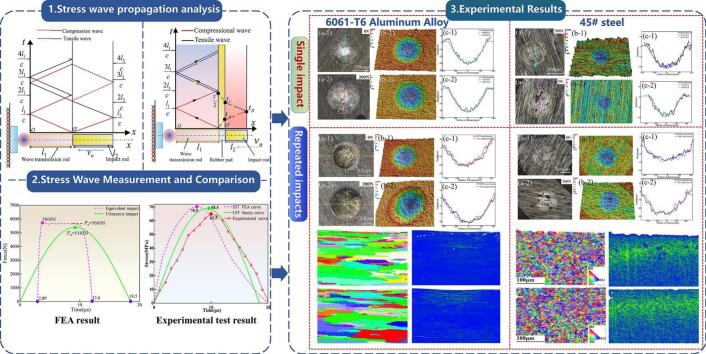

## Nomenclature

A: Material constant of Johnson-Cook model(325 MPa) (714 MPa)
*A*_0_: Vibration amplitude(10 μm)
B: Material constant of Johnson-Cook model(250 MPa) (600 MPa)
C: Material constant of Johnson-Cook model(0.02) (0.03)
*c*: Wave velocity
*c_p_*: Velocity of wave propagation in Solid(5170 m/s)
*c_r_*: The velocity of wave propagation in rubber(215 m/s)
*E_T_*: Energy within single-cycle ultrasonic impact(0.168 J)
*E_k_*: Equivalent impact energy(0.084 J)
*_EY_*: Young's modulus
*_Fu_*: Max-contact force in ultrasonic impact theory (5385 N)
*_Fe_*: Max-contact force in equivalent impact theory (5385 N)
*f*: Frequency of ultrasonic vibration(27 kHz)
*g*: Gravitational acceleration(9.8 m/s^2^)
*h*: Drop height(581 mm)
_Δ_*_h_*: Height of rubber pad(1 mm)
*_l_*_2_: Impact rod length(23.9 mm)
*_l_*_1_: Wave transmission rod length(47.8 mm)
*_mn_*: Impact rod equivalent mass(14.735 g)
*_ρ_*: Density of horn material(7850 kg/m^3^)
*ω*: Angular velocity(169.56e3 rad/s)
*φ_0_*: Initial phase angle(0°)
*_λ_*: Wave length in 45**#** steel(191 mm)
_σ*_: Material constant of Johnson-Cook model
*_σ_*: Stress wave intensity
*_m_*: Temperature softening coefficient(1.5)
*_n_*: Strain-hardening exponent(0.29) (0.25)
*_R_*: Impact head radius(7 mm)
*_rn_*: Wave transmission rod radius(5 mm)
*_r__w_*: Impact rod radius(5 mm)
*S*: Cross-sectional area(78.5 mm^2^)
*_Tr_*: Melting temperature(2000 ℃)
*_Tm_*: Reference temperature(20 ℃)
*_T_*: Cycle time(37 μs)
*_tc_*: Total travel time in impact rod(9.25 μs)
_Δ_*_t_*: Total propagation time on rubber pad (9.25 μs)
*t*: Time
*V*: Horn volume
*_v_*: Velocity of ultrasonic vibration(1.69 m/s)
*_vn_*: Impact rod velocity(3.38 m/s)
*_vw_*: Wave transmission rod velocity(1.69 m/s)
*_w_*: Energy density
*_x_*: Vibrating particle coordinate
*_y_*: Displacement of ultrasonic vibration
*_σin_*: Incident wave stress
*_σr_*: Reflected wave stress
$\dot{\varepsilon }$: Plastic strain rate
$\overset{˙}{\varepsilon_{0}}$: Reference plastic strain rate
*_μ_*: Poisson's ratio(0.3) (0.33)

## 1 Introduction

Ultrasonic impact treatment (UIT) is a novel surface processing technology that enhances component surface quality and fatigue performance by superimposing a high-frequency (typically 20–40 kHz) impact load driven by ultrasonic waves onto a static load [1], [2]. The dynamic load from the impact head interacts with the static load, inducing severe plastic deformation on the workpiece surface. This process generates high-density dislocations and high-amplitude residual compressive stresses [3], [4]. The plastic deformation induced by repeated impacts causes minute peaks in the surface material to flow toward troughs, homogenizing the microstructure and forming a surface-hardened layer with lower roughness [5], [6], thereby achieving more complete surface integrity. Research indicates that the high strain rate induced by ultrasonic impact significantly enhances microhardness near the surface-treated zone [7], [8]. The introduced residual compressive stress(RCS) field effectively suppresses fatigue crack initiation and propagation, with RCS magnitude increasing within a certain range as the number of impacts rises [9], [10]. RCS can effectively enhance a component's fatigue resistance and stress corrosion resistance, thereby extending its service life [11], [12]. To better leverage these numerous advantages, research on the mechanism of ultrasonic impact action has gradually become a focal point [13], [14].

With advancements in computational mechanics and experimental testing techniques, researchers have established diversified experimental testing platforms and finite element analysis (FEA) methods to extensively explore the coupled interaction mechanism between static and dynamic loads during ultrasonic impact. [15], [16] Unal et al. [17] investigated ultrasonic impact testing on AISI 1050 medium-carbon steel and found that increasing static loads improves workpiece surface roughness, significantly enhancing the component's high-stress-low-cycle fatigue performance. However, excessively high static loads conversely increases roughness, indicating the need for optimal parameter selection., indicating the existence of an optimal static load interval, where surface strengthening effects and surface integrity must be balanced through parameter optimization. Zhang et al. [18] employed a spring-damper model to simulate equivalent loads during ultrasonic impact, identifying a critical amplitude or static loads required to maintain tool dynamic stability, providing crucial theoretical guidance for process parameters selection,which lays the foundation for establishing more refined dynamic equivalent load models. Meng et al. [19] developed quasi-static and dynamic models for ultrasonic impact. Their experiments revealed that residual stress depth in 6061-T6 Aluminum Alloy increases with loads magnitude, with dynamic load dominating the depth evolution process, thereby offering experimental guidance for investigating the internal energy transfer mechanisms induced by impact loading. Guo et al. [20] established single and double impact FEA models for pins to approximate elastoplastic deformation behavior during ultrasonic impact,providing a novel framework for designing equivalent impact experiments. Seok et al. [21]proposed a displacement based FEA method and experimentally validated its high predictive accuracy for residual stress distribution after single and multiple ultrasonic impact cycles, demonstrating the feasibility of constructing accurate impact energy input models.

Although extensive research has explored the optimization of ultrasonic impact process parameters through experimental methods and FEA [22], [23], most studies have focused on the effects of static load [24], [25], [26], spindle speed [26], [27], ultrasonic frequency and amplitude [28], [29], [30], friction type combinations [31], [32], combination of techniques [14], [33], and material categories on fatigue resistance or wear resistance [34], [35]. Given the periodic and highly transient nature of ultrasonic impact, systematic research remains needed on fundamental issues such as the mechanical behavior and interaction mechanisms between the impact head and workpiece within a single-cycle.

This paper analyses the energy transfer mechanism of stress waves in ultrasonic impact [36], [37] and proposes an experimental method for equivalently simulating the impact behavior of ultrasonic impact within a single-cycle by exciting stress wave through one-dimensional rod collisions. The synergistic effects of static and dynamic loads during ultrasonic impacting are comprehensively considered to establish a research framework that integrates an equivalent theoretical model with numerical simulations and experimental validation. This approach verifies the accuracy and applicability of the proposed model. Section 2 elaborates on modeling method for dynamic and static loads. Based on the principle of energy equivalence, an equivalent application method for dynamic load during a single-cycle ultrasonic impact is innovatively proposed. By analysing the propagation patterns of ultrasonic waves within the horn, a quantitative conversion relationship for equivalent dynamic load is established, providing theoretical foundations for FEA modeling and impact test parameter design. Section 3 employs FEA to model the elastoplastic processes of single-cycle ultrasonic impact and equivalent impact treatment(EIT). It focuses on comparing the loads history between the impact head and workpiece, the contour morphology of the processed region, and the distribution characteristics of residual stress fields. Section 4 conducts experimental studies on single-point ultrasonic impact and equivalent impact using an ultrasonic impact machine and a self-built equivalent impact test platform. Section 5 employs laser confocal microscopy and X-ray stress measurement instruments to precisely characterize the microstructure and residual stress in the processed zone, followed by quantitative comparison and error analysis between experimental and simulated results. Section 6 provides a comprehensive summary of this research, highlighting the advantages of the energy equivalence-based equivalent impact method in analysing ultrasonic impact transient processes and outlining future research directions. The findings offer a viable approach for investigating ultrasonic impact mechanisms, providing theoretical guidance and technical support for process optimization, and contribute to the advancement of this specialized machining technology.

## 2 Theoretical model analysis

This section first calculates the energy of a single ultrasonic impact cycle based on mechanical wave theory [38].By taking the impact pulse width, stress wave amplitude, and waveform as the core equivalence conditions, a corresponding equivalent impact model is established. According to stress wave propagation theory and classical physical formulas, these equivalence conditions are quantitatively analyzed. The proposed model provides a key theoretical and analytical foundation for systematically investigating the effects of ultrasonic impact under different process parameters.

### 2.1 Ultrasonic impact theoretical modeling

**Fig. 1:**
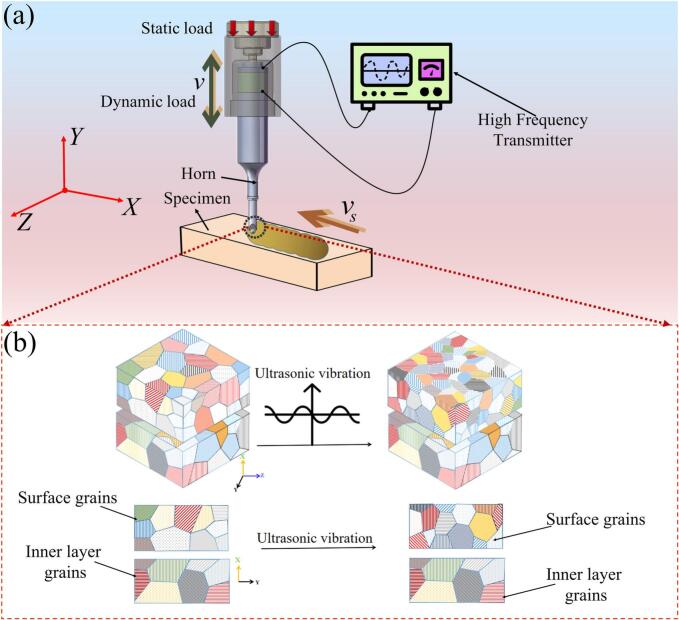
Schematic diagram of working principle of ultrasonic impact. (a) Schematic diagram of ultrasonic impact. (b) Grain refinement diagram.

The primary feature of UIT is the application of a relatively stable static preload to the workpiece surface via the impact head. Secondly, high-frequency electrical signal generated by an ultrasonic generator is converted into mechanical vibration of the same frequency via a transducer [39], [40]. This vibration is then transmitted as mechanical waves through an amplitude-amplifying rod, where the amplitude is amplified, as shown schematically in Fig.1(a). This amplified vibration drives the impact head to apply dynamic load to the workpiece surface to gain an optimal microstructure and mechanical properties in the surface layer [41], [42], as shown schematically in Fig.1(b).The motion of the horn during ultrasonic impact is a manifestation of high-frequency, one-dimensional elastic stress waves propagating directionally along its axis. Upon reaching the tip, the mechanical energy of these waves drive the impact head, ultimately generating dynamic loads to the workpiece. Notably, due to the inherent high-frequency nature of ultrasonic vibration, the impact head acts on the workpiece through rapid, periodic impacts, distinguishing it from traditional static processing methods. The extreme brevity of each cycle(typically under 50 μs) poses a significant challenge for accurate measurement with conventional methods. Therefore, clarifying the action patterns within a single-cycle remains a crucial prerequisite for revealing the working mechanism of UIT. This paper consequently focuses on the analysis and verification of stress wave propagation patterns during a single impact cycle.

Assuming the ultrasonic source undergoes harmonic oscillation at frequency *f*, according to one-dimensional elastic wave theory, the generated elastic stress waves propagate along the horn at wave velocity *c*. The equation of motion for any particle on the horn is given by Eq. (1), (2). Differentiating this equation yields the propagation particle velocity at any given time [43], [44]:

(1) $$y=A_{0}\sin\left[\omega \left(t-\frac{x}{c}\right)+\phi_{0}\right]$$

(2) $$v=\frac{\mathrm{dy}}{\mathrm{dt}}=\omega A_{0}\cos\left[\omega \left(t-\frac{x}{c}\right)+\phi_{\text{0}}\right]$$

After the system stabilizes, the mechanical energy density contained within a unit volume of the boom can be expressed by the formula:

(3) $$w=\frac{\mathrm{dE}}{\mathrm{dV}}=\rho A_{0}^{2}\omega^{2}\sin^{2}\left[\omega \left(t-\frac{x}{c}\right)\right]$$

From Eq. (3), it can be seen that the energy density of the propagating axially stress wave within the ultrasonic impact horn exhibits a sinusoidal distribution at any instant. Considering the periodic nature of the ultrasonic impact, integrating the stress wave energy density over any vertical cross-section of the horn within one cycle yields the mechanical energy transmitted through the horn to the workpiece during that cycle by Eq. (4):

(4) $$E_{T}=\int_{0}^{T}wdV=\int_{0}^{T}\mathrm{wSc}dt=\int_{0}^{T}\rho A_{0}^{2}\omega^{2}Sc\sin^{2}\left[\omega \left(t-\frac{x}{c}\right)\right]dt=\frac{\rho A_{0}^{2}\omega^{2}S\lambda }{2}$$

Taking the volume element as the object by Eq. (5), according to the impulse theorem, it can be concluded that:

(5) σSdt=ρScdtv

The relationship between the stress wave intensity and the particle velocity can then be obtained as follows by Eq. (6)[45]:

(6) σ=ρcv

That the essence of ultrasonic impact is the transmission of one-dimensional sinusoidal elastic stress waves through the amplitude rod to the impact head. These waves, characterized by a period of *T = λ/c* and a total energy of *E_T_* per-cycle, comprise alternating compressional and tensile half-cycles. Critically, the compressional phase, carrying a mechanical energy of 1/2 *E_T_*, compel the impact head to exert periodic impact forces on the workpiece. Therefore, this paper proposes an equivalent experimental method: triggering a one-dimensional elastic stress wave of specific intensity and width through a single impact, it approximates the stress wave transmitted by ultrasonic impact within a single-cycle. This enables the sequential separation and equivalent reproduction of the high-frequency dynamic impact process. This approach addresses bottlenecks such as the difficulty in controlling ultrasonic impact processes and characterizing the history parameters of elastoplastic deformation. This approach successfully decouples the complex high-frequency process, enabling the separate study of its dynamic component. It thereby overcomes critical problems in process control and parameter characterization, offering new research perspectives and quantitative analysis methods for optimizing ultrasonic impact process parameters and investigating the coupling mechanism between dynamic and static loads in ultrasonic impact.

### 2.2 Establishment of an equivalent model for ultrasonic impact

**Fig. 2:**
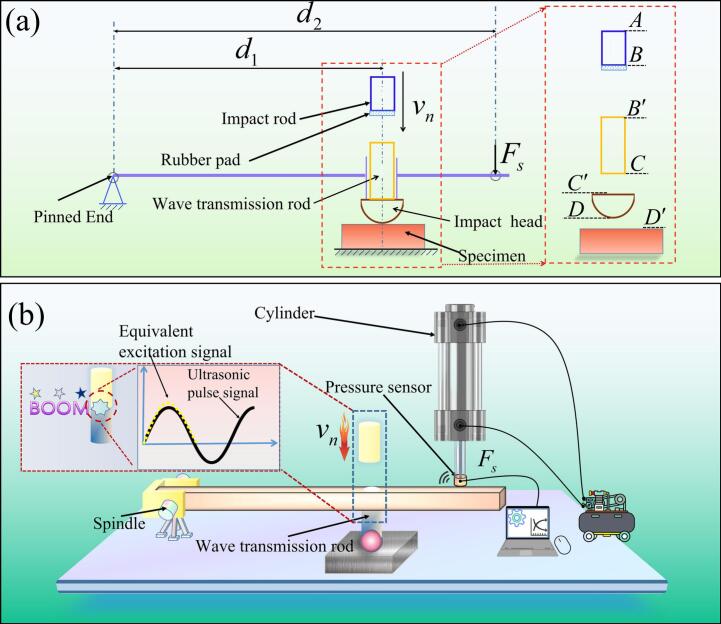
Principle and schematic diagram of equivalent impact. (a) Principle diagram of equivalent impact. (b) Schematic illustration of equivalent impact.

The equivalent impact test platform designed in this paper simulates a single-cycle ultrasonic impact stress wave via the collision of two one-dimensional rods. Its fundamental principle is illustrated in Fig. 2. First, the cylinder applies the required static load *F_s_* to the impact head via a lever mechanism. Subsequently, the impact rod strikes the wave transmission rod at a predetermined velocity *v_n_*, generating a stress wave of corresponding intensity. This wave propagates along the wave transmission rod to the impact head and ultimately into the workpiece.

When the impact rod strikes the stationary wave transmission rod at velocity *v_n_*, the contact rod ends must satisfy the boundary condition of equal velocities [38]. When both rods share identical material and diameter, the post-impact velocity of their particles satisfy by Eq. (7):

(7) $$v_{w}=\frac{v_{n}}{2}$$

Based on Eq. (7), the intensity of the stress wave generated when the impact rod strikes the wave transmission rod and the required free-fall height can be calculated Eq. (8):

(8) $$h=\frac{v_{n}^{2}}{2g}$$

### 2.3 Analysis of stress wave propagation pathways

Based on one-dimensional elastic wave theory [46], [47], and adhering to the assumptions of small deformation and material homogeneity, transverse effects are neglected. When the BB' surfaces come into contact, the impact commences. At this point, the BB' surfaces undergo compression, converting the kinetic energy of the impact rod into elastic potential energy and generating a pair of compressional longitudinal waves propagating toward the distal ends of both rods. The plane wave equation they satisfy can be described as by Eq. (9)[48]:

(9) $$\frac{\partial^{\text{2}}u}{\partial t^{2}}=c_{p}^{2}\frac{\partial^{\text{2}}u}{\partial x^{2}}$$

Eq. (9) describes the relationship between the displacement *u* of a particle and time *t* and position *x*. This equation characterizes the dynamic behavior of the displacement field of elastic waves propagating through an elastic medium.Where *f* represents a right travelling wave and *g* represents a right travelling wave.

By applying mathematical analysis method, an explicit analytical expression for the equation is established, with the specific formula by Eq. (10):

(10) $$u\left(x,t\right)=f\left(x-c_{p}t\right)+g\left(x+c_{p}t\right)$$

When a stress wave propagates to a pinned end, displacement at the end surface is forced to zero. At this point, it is assumed that no displacement occurs at this end surface. In this case, the boundary conditions are given by Eq. (11):

(11) $$u\left(\text{0},t\right)=f\left(-c_{p}t\right)+g\left(c_{p}t\right)=\text{0}$$

Simultaneously analysing the stress on the pinned end surface yields by Eq. (12):

(12) $$\sigma_{\mathrm{in}}=E_{Y}\frac{\partial u}{\partial x}=E_{Y}f^{\prime }\left(x-c_{p}t\right)$$

(13) $$\sigma_{r}=E_{Y}g^{\prime }\left(x+c_{p}t\right)=E_{Y}f^{\prime }\left(-x-c_{p}t\right)$$

At the pinned end boundary (*x* = 0), substituting into Eq. (13) yields the reflected wave stress at this location:

(14) $$\sigma_{r}\left(\text{0,}t\right)=E_{Y}f^{\prime }\left(-c_{p}t\right)=\sigma_{\mathrm{in}}\left(0,t\right)$$

From Eq. (14), it can be seen that when the incident wave propagates to the pinned end and forms a reflected wave through reflection, although the stress direction reverses, the propagation direction also reverses. Therefore, the stress wave state remains unchanged and still aligns with the incident wave. For the free end, the stress is forced to zero. The summary of both cases is shown in Table 1.

**Table 1:** End-Face reflection summary table.

Boundary type	Boundary condition	Reflected waveform	Stress wave variation
Pinned end	*u*(0,*t*) = 0	*g*(*x + c_p_t*) *= -f*(*x-c_p_t*)	Compressional wave → Compressional wave
Free end	*σ*(0,*t*) = 0	*g*(*x + c_p_t*) *= f*(*x-c_p_t*)	Compressional wave → Tensile wave

Based on the aforementioned stress wave propagation theory[49], the propagation path of the stress wave during the collision is illustrated in Fig. 4.

**Fig. 3:**
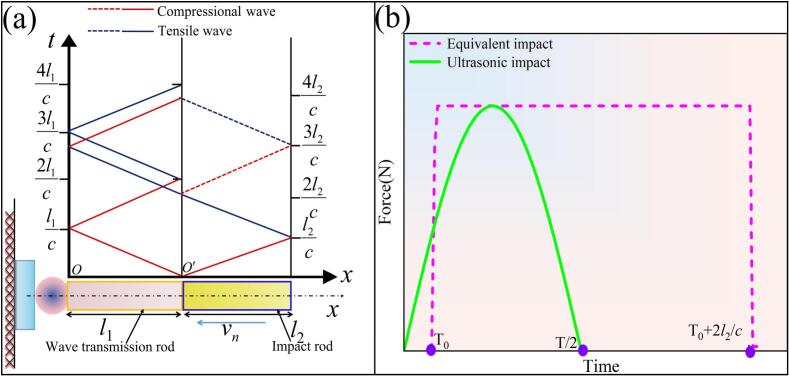
(a) Equivalent physical plane diagram of stress waves propagation across rods. (b) Theoretical results for stress waves amplitude before correction.

As shown in Fig.3 (a), when the right-traveling compressional wave reaches the end and reflects as a tensile wave, it causes the separation of the two rods upon arriving at the OO' end face. Therefore, the effective duration of the equivalent impact process satisfies the following condition by Eq. (15):

(15) $$t_{c}=\frac{2l_{\text{2}}}{c_{p}}$$

Furthermore, the equivalent impact process can be regarded as that the impact rod inputs a rectangular stress wave with intensity *ρcv_w_* and width 2 *l*_2_*/c_p_* into the wave transmission rod [50]. The impact process of ultrasonic impact, on the other hand, inputs a half-sine stress wave with amplitude *ρcωA*_0_ and width *T*/2. As shown in Fig.3 (b), the propagation path of the stress wave input at point B' in the transmission rod exhibits a certain similarity to that of the ultrasonic impact. Through reasonable parameter configuration and correction, the equivalent reproduction of a single ultrasonic impact cycle can be achieved.

### 2.4 Equivalent conditions for impact models

To achieve high-precision reproduction of single-cycle ultrasonic impact processes through equivalent impact models, strict equivalence conditions must be satisfied regarding the mechanical energy released during the impact process, the intensity of stress wave, and their propagation patterns. The procedure for establishing these equivalence conditions is as follows.

1) Controlling the velocity *v_n_* of the impact rod to generate stress wave of the same amplitude as ultrasonic impact satisfies the requirement by Eq. (16):

(16) $$v_{n}=2A_{\text{0}}\omega$$

2) The impact rod delivers the same mechanical energy to the wave transmission rod input and the ultrasonic impact process (half cycle), satisfying Eq. (17):

(17) $$\frac{1}{2}m_{n}v_{n}^{2}=\frac{1}{2}\pi \rho r^{2}l_{2}v_{n}^{2}=\frac{1}{2}E_{T}=\frac{\rho A_{0}^{2}\omega^{2}S\lambda }{4}$$

According to Eq. (16) and Eq. (17), the length of the impact rod is calculated as *l_2_* = *λ*/8, with the corresponding stress wave loading pulse width being *T*/4.

**Fig. 4:**
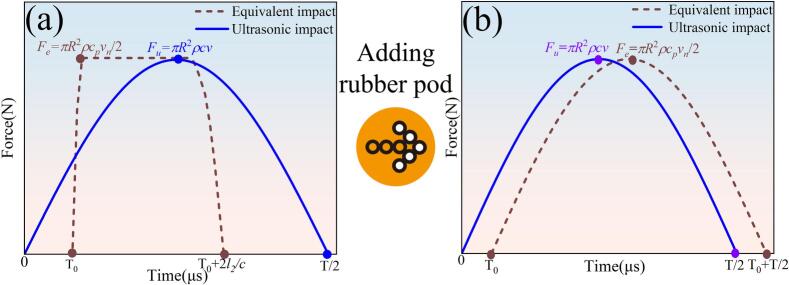
Theoretical results for stress wave amplitude. (a) Results before correction. (b) Results after correction with rubber pod addition.

As shown in Fig. 4(a), the stress wave input to the wave transmission rod by the impact rod should exhibit the same time history curve as the ultrasonic impact process. Although both stress waves share the same amplitude, the stress wave from the equivalent impact is a rectangular wave with a width of *T*/4, whereas the loading wave from ultrasonic impact is a sinusoidal wave with a width of *T*/2. A certain discrepancy still exists between the two, necessitating further waveform refinement.

**Fig. 5:**
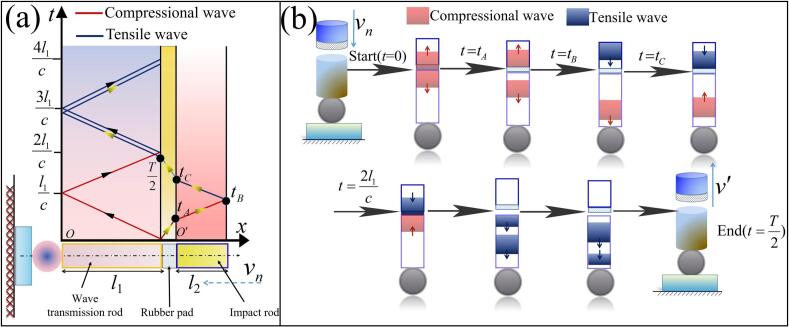
Stress waves propagation pattern. (a) Equivalent physical plane diagram of stress waves propagation. (b) Propagation time diagram of stress waves in the rod and rubber pad.

Considering the corrective effect of spacers on waveform in Hopkinson bar experiments [51], [52], a rubber pad with identical cross-section and thickness Δ*h* is added at the interface between the impact rod and the wave transmission rod. This extends the duration of stress wave action while refining the original rectangular waveform. As shown in Fig. 5(a), the propagation path of the stress wave within both the rubber pad and the impact rod is twice of their respective lengths. Consequently, the propagation time of the stress wave within the rubber pad and impact rod satisfies the following relationship Eq. (18):

(18) $$\Delta t=\frac{2\Delta h}{c_{r}}+\frac{\text{2}l_{2}}{c_{p}}=\frac{T}{2}$$

Since the propagation speed of stress wave in rubber is only 215 m/s, significantly lower than that in 45**#** steel, the calculated thickness and mass of the rubber pad are both much smaller than those of the impact rod and wave transmission rod. Therefore, it does not cause significant energy loss when correcting the waveform characteristics and pulse width of the stress wave. The final stress wave propagation after model correction is shown in Fig. 5.

## 3 Finite element analysis

Based on the theoretical framework of the equivalent model, this section establishes two axisymmetric finite element models [42]: one for the equivalent impact (impact rod--wave transmission rod--impact head--workpiece assembly) and another for ultrasonic impact (horn--impact head--workpiece assembly). Both models utilize a dynamic implicit [53], [54] analysis method to compare dynamic response characteristics and impact effects. The Johnson-Cook [55] constitutive model was employed to capture the material strain rate sensitivity. The accuracy of the equivalent model was subsequently validated through a multi-faceted analysis of simulation results, including time history curve, impact pit morphology, and residual stress.

### 3.1 Model parameter selection

The Johnson-Cook constitutive model, presented in Eq. (19)[56], was implemented in the simulation. The material parameters for 6061-T6 Aluminum Alloy [57], [58] and 45**#** steel [9], [59] are provided in Table 2. A axisymmetric computational model of the specimen (Φ60 mm × 15 mm) was established to reduce computational expense. The initial condition was defined by applying the impact rod velocity derived from Eq. (7) to simulate a single-point impact.

(19) $$\sigma^{*}=\left(\text{A}+\text{B}\varepsilon^{n}\text{)(1}+\text{Cln}\frac{\dot{\varepsilon }}{{\dot{\varepsilon }}_{\text{0}}}\text{)(1 -}\;{\left[\frac{T-T_{r}}{T_{m}-T_{r}}\right]}^{m}\right)$$

**Table 2:** C constitutive model parameter table for 6061-T6 Aluminum Alloy and 45# steel.

Material	A (MPa)	B (MPa)	*n*	C	*m*	Density (kg/m^3^)	Elastic modulus (MPa)	Poisson's ratio
6061-T6	325	250	0.29	0.02	1	2730	69,000	0.33
45**#**steel	714	600	0.25	0.03	1.5	7850	210,000	0.3

### 3.2 Ultrasonic impact and equivalent impact FEA modeling

**Fig. 6:**
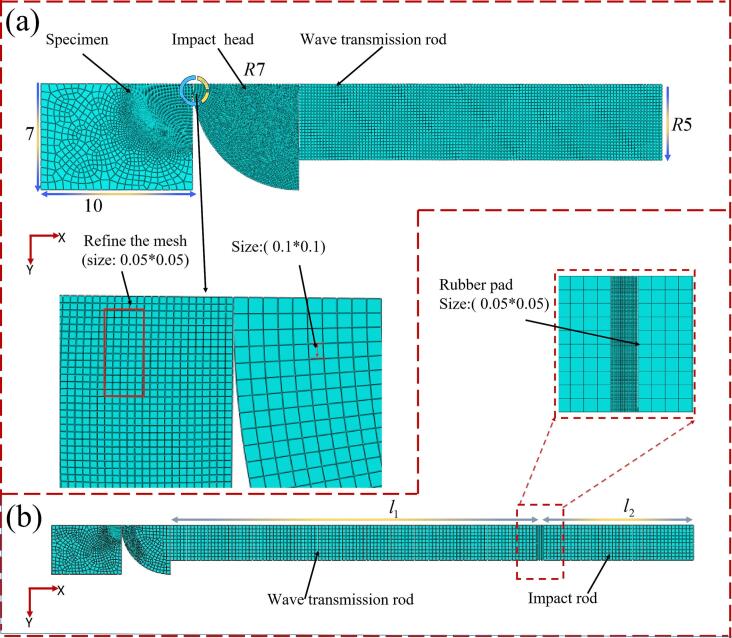
Schematic diagram of FEA model. (a) Schematic of ultrasonic impact FEA. (b) Schematic of equivalent impact FEA.

This study establishes axisymmetric simulation models for ultrasonic and equivalent impact in ABAQUS [60], [61], based on the parameters of a 27 kHz ultrasonic system, as illustrated in Fig. 6.

Fig. 6(a) shows the ultrasonic impact model. A 27 kHz sinusoidal excitation was applied to the horn's front end, resulting in a no-load amplitude of 10 μm at its tip.

Fig. 6(b) presents the equivalent impact model. According to the calculations in 2.2, 2.4, a single-cycle at 27 kHz and 10 μm amplitude carries an energy of 0.084J, which yields an impact rod velocity (*v_n_*) of 3.38 m/s. Both the impact and transmission rods were set as 45# steel. Since these components experience negligible plastic deformation in practice, they were defined as purely elastic to simplify the model. The contact parameters between the impact head and the workpiece, as well as the boundary fixation using the XSYMM symmetry condition, were kept consistent with the ultrasonic impact model.

A hard silicone rubber spacer with a thickness of 1 mm was inserted between the two rods, as determined by Eq. (18), to ensure equivalent stress wave transmission duration. The FEA model was discretized using a structured CAX4R mesh with an element size of 0.05 mm.

### 3.3 Analysis of simulation results

**Fig. 7:**
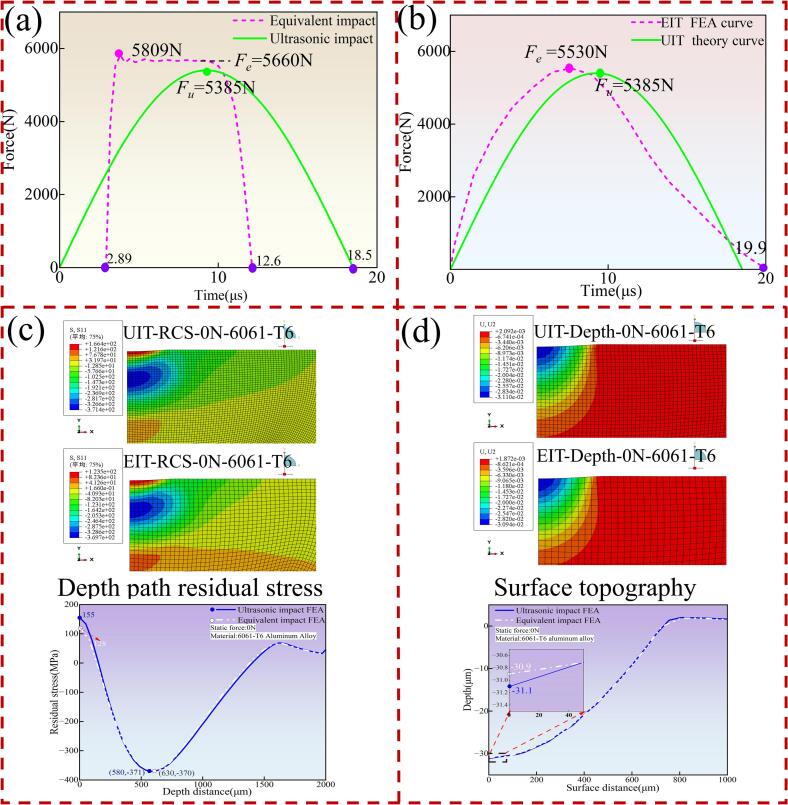
FEA results. (a) Impact excitation pulse width. (b) Impact excitation pulse width with rubber pad. (c) Residual stress contour map. (d) Deformation contour map.

Fig. 7(a) presents the simulated equivalent impact loads under the conditions determined by Eq. (16), (17). The loads profile approximates a rectangular waveform, featuring an initial overshoot [[62], [63]]to 5809 N before stabilizing at 5660 N. The stabilized amplitude shows a mere 4.8% relative error compared to the theoretical value of 5385 N. The pulse width of 9.71 μs deviates by 4.7% from the theoretical T/4 value (9.25 μs). As anticipated by the analysis in Fig. 7(a), this waveform remains distinct from the sinusoidal input of the ultrasonic impact.

Guided by the theory in Section 2.4 and Eq. (18), a 1 mm thick rubber pad was incorporated between the rods to refine the waveform. The resulting stress wave signal is shown in Fig. 7(b). A comparison with the theoretical input signal (in green) reveals errors of 7.7% in pulse width and 2.6% in amplitude. The two load curves demonstrate strong consistency in pulse width, amplitude, and overall morphology, thereby effectively validating the theoretical approach and providing a reliable foundation for subsequent experimental design.

The simulation results for residual stress and deformation of 6061-T6 Aluminum Alloy under ultrasonic impact and equivalent impact (0 N) are detailed in Fig. 7(c–d), respectively. The maximum RCS are 371 MPa and 370 MPa, with a negligible deviation of 0.27%. These stress peaks are located at similar depths near 600 μm, and their depth-wise distributions correlate well. The maximum deformation depths are 31.1 μm and 30.9 μm, deviating by only 0.65%, and the pit diameters differ by approximately 2%. The high degree of consistency in these key metrics confirms the effect equivalence between the two simulation models.

To verify the general applicability of the equivalent impact model established for the system, this study comprehensively evaluated the reliability of its simulation results through a multi-parameter analysis. This includes the following two types of typical conditions: 6061-T6 Aluminum Alloy under a static load of 200 N, and 45**#** steel under static load of 0 N and 500 N. The comparison results between the simulation and experiments for the above three conditions are detailed in Appendix 1 of this paper.

A comprehensive analysis of these three sets of comparative figures reveals that even with significant variations in static load level and across materials, the proposed equivalent impact model exhibits a high degree of detailed similarity with the single-cycle ultrasonic impact simulation results. This similarity manifests in multiple key physical quantities: both the distribution patterns and magnitudes of residual stresses, as well as the surface contour features formed after impact, exhibit numerical deviations not exceeding 4.8% (max-RCS) and 4.5% (depth of pit).

Thus, the simulation results confirm the theoretical validity and practical applicability of the proposed equivalent model. This confirmation paves the way for the subsequent experimental validation.

## 4 Experimental validation

This section first presents a detailed description of the structure and functional modules of the self-developed equivalent impact experimental platform. Subsequently, the measurement equipment required for the experiments and their key technical parameters are systematically introduced.

### 4.1 Experimental materials and apparatus

To validate the practical effectiveness of the theoretical framework proposed in this study, reliance solely on FEA results is insufficient. Therefore, following the methodology described in Section 3, both equivalent impact and ultrasonic impact experiments were conducted on 6061-T6 Aluminum Alloy and 45**#** steel to verify the model’s predictive accuracy. For experimental consistency and ease of clamping, all specimens were fabricated as rectangular blocks with uniform size of 60 mm **×** 60 mm **×** 15 mm. A tungsten carbide ball with a radius of 7 mm was employed as the impact head, identical to that used in the ultrasonic impact system.

**Fig. 8:**
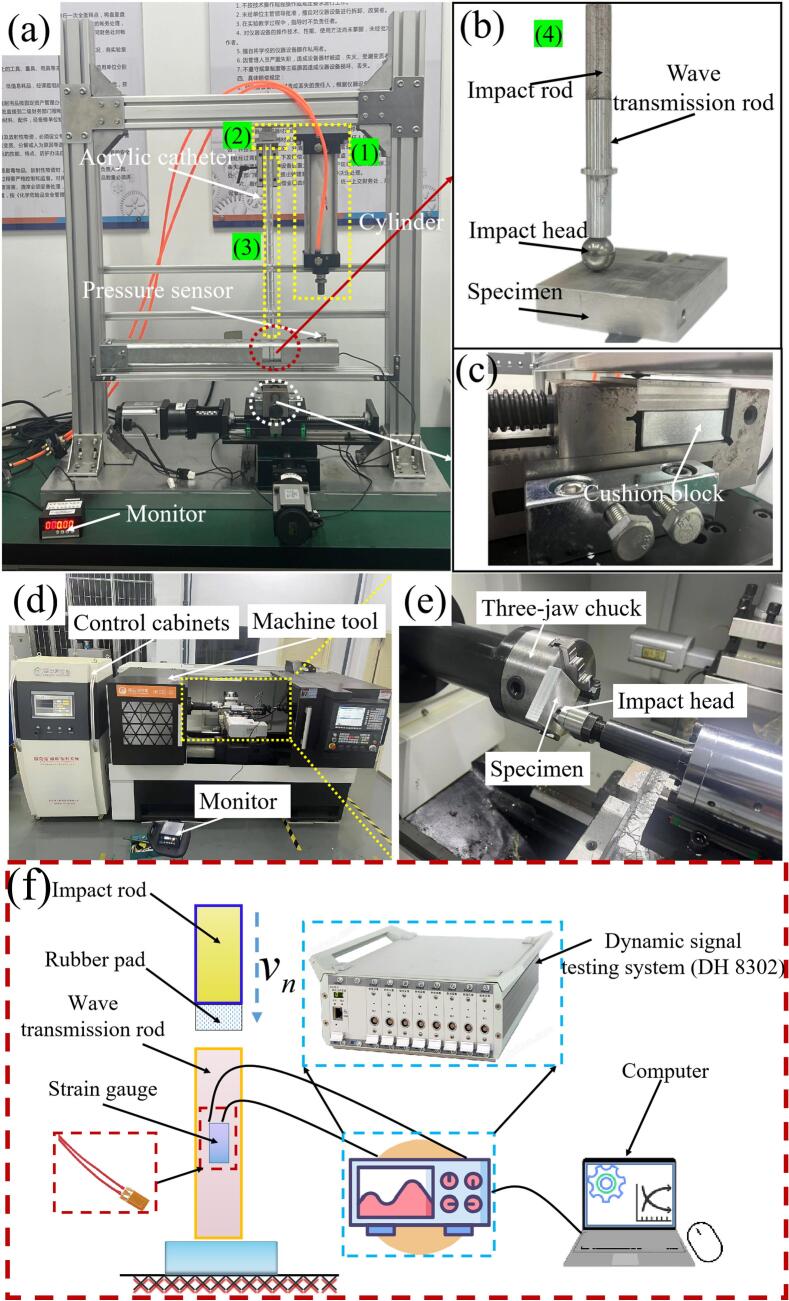
EIT and UIT test apparatus diagram. (a) Test apparatus. (b) Energy transfer device. (c) Specimen clamping. (d) Ultrasonic impact CNC lathe. (e) UIT local magnification.. (f) Stress wave measurement.

The experimental apparatus, shown in Fig. 8(a), was independently developed based on the proposed equivalent model. It consists of four core modules: (1) Static load module: applies the required static load via a cylinder and lever mechanism. (2) Positioning module: adjusts the initial height of the impact rod to set the desired impact energy. (3) Guide module: utilizes customized acrylic tubing to control the descent path with a multi-hole array design to achieve dynamic air pressure balance, effectively eliminating air damping during the impact rod's free fall. (4) Energy transfer module: the dynamic coupling and spatial configuration of the impact rod, wave transmission rod, ball head, and workpiece are detailed in the schematic diagram of Fig. 8(a–c).

The equivalent impact experiments were performed under ambient temperature conditions. The experimental parameters were derived from the equivalent model, with the static load precisely applied by regulating the cylinder pressure and the free-fall height of the impact rod determined by the impact energy. Simultaneously, material impact test was conducted on an ultrasonic impact lathe platform, as illustrated in Fig. 8(d) quantitative error analysis between the two experimental sets was performed, encompassing the micro-morphology, residual stress, microhardness of impact pit, and microstructural characteristics of the affected layer. This comprehensive comparison revealed a definitive correlation between ultrasonic impact and equivalent impact experiments.

As shown in Fig. 8(e), with the spindle stationary, the ultrasonic head moves along the machine tool's axial direction (Z-axis) and applies a single-point impact with an amplitude of 10 μm to the end face of the workpiece.The energy of a single-cycle ultrasonic impact was calculated using Eq. (5) and Eq. (10). To ensure consistency with this calculated energy[64], the impact rod's drop height was set to 128 mm for the subsequent EIT experiments.

### 4.2 Experimental testing equipment

A cross-scale characterization job was implemented for specimens of both materials treated with equivalent impact and ultrasonic impact processes respectively. Quantitative analysis of key response variables was conducted with the following high-precision instruments: Stress wave signal during single impact event was acquired with a dynamic signal testing system (DH 8302) at 1 MHz. Strain gauge[65], [66] (BE120-3AA-P100 120 Ω) was bonded axially to the wave transmission rod with 502 adhesive and wired in a quarter-bridge configuration without temperature compensation,the measurement diagram is shown in Fig. 8f.. Laser confocal microscopy (KEYENCE VK-X1000) was employed to measure the surface topography of the impact indentations. Residual stress was determined by an X-ray stress analyzer (Proto LXRD HDS-I, Cr-Kα radiation, diffraction) using the sin^2^*ψ* method. The microhardness of the surface hardened layer was measured at the indentation center using a Vickers microhardness tester (Buehler FALCON 1) with a load of 0.1 N. Additionally, Electron Back Scatter Diffraction (EBSD) analysis was performed using a scanning electron microscope (Carl Zeiss Crossbeam 550) to observe impact-induced grain changes.

## 5 Results and discussion

This section first obtained the stress wave time-history curve of a single impact through strain gauge measurements. Single and multiple impact experiments were then conducted on 6061-T6 Aluminum Alloy and 45**#** steel respectively. From multiple perspectives—including three-dimensional impact crater morphology, microhardness distribution, and residual stress fields—the accuracy of the proposed equivalent impact model was systematically validated.Further multiple-impact experiments demonstrated that the model is also capable of effectively simulating the cumulative effects of ultrasonic impact, showing good applicability across different materials and processing parameters.

### 5.1 Comparison of stress wave history curve

**Fig. 9:**
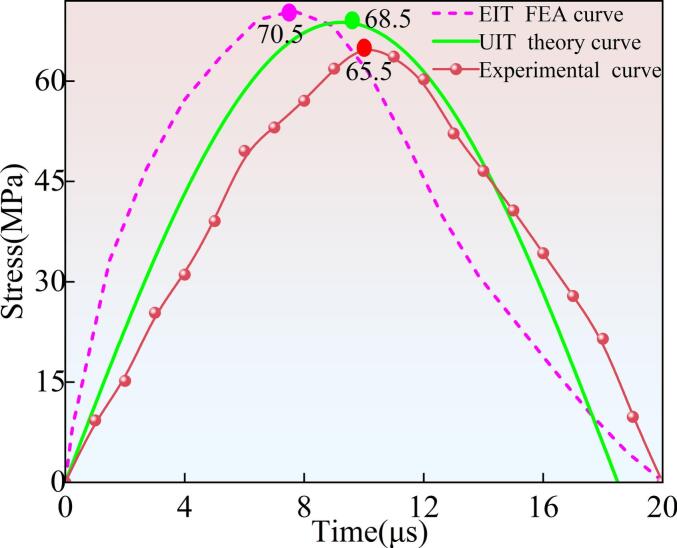
Stress wave experimental result curve.

Prior to comparing the equivalent impact effects, it is necessary to verify the consistency between the input waveform and the single-cycle ultrasonic impact signal through single impact stress wave measurement. Following the experimental methodology detailed in section 4.2 Fig. 9 presents the stress wave over time measured during the equivalent impact process. A comparison with the theoretical curve reveals a maximum amplitude error of 4.4% and a pulse width difference about 1 μ s (approximately 5.4%). The measured stress wave curve shows good agreement with theoretical predictions, thereby validating the equivalence of the present experimental scheme in simulating the ultrasonic impact from the perspective of stress wave characteristics.A key advantage of the methodology is its capacity to reproduce frame-by-frame the high-frequency ultrasonic impact process, akin to viewing it in slow motion. This capability enables an in-depth investigation into the coupling mechanism of dynamic and static loads and the surface deformation mechanism in ultrasonic impact.

It is noteworthy that the measured stress wave exhibits a certain degree of distortion compared to the theoretical half-cycle sinusoid: the response during the rising phase is lower than the theoretical value, while it exceeds the theoretical value during the decaying phase. This systematic deviation is attributed to two primary factors: firstly, the theoretical model assumes ideal contact plane and boundary constraints, whereas the contact planes are not completely parallel and the end of the wave transmission rod is not completely constrained either in actual experiment[67], leading to an extended contact duration; secondly, the propagation of stress wave in the rod is subject to geometric dispersion induced by lateral inertia due to the three-dimensional Poisson effect [68], [69], which further aggravates the waveform distortion.

In the present theoretical model, stress wave propagation is approximately treated as one-dimensional axial propagation. However, the actual ultrasonic impact system possesses finite lateral dimensions, and therefore certain transverse inertia effects and higher-order vibration modes may be excited under high-frequency transient impact conditions. According to the Pochhammer–Chree dispersion theory [70], different frequency components in cylindrical structures propagate at different velocities. Although the wave propagation process in the present study is dominated by the 27 kHz frequency component, some higher-frequency components may gradually separate from the main wave packet during propagation, resulting in peak delay and waveform broadening in the experimentally measured signal. In addition, microscopic surface roughness and local non-uniform contact regions inevitably exist at the actual contact interface, which can further induce stress-wave scattering and additional energy dissipation. Consequently, the experimentally measured peak stress is slightly lower than the theoretical prediction.

Meanwhile, due to the relatively short stress-wave propagation distance and the small cross-sectional dimension of the present impact system (much smaller than the wavelength), the accumulated effect of geometrical dispersion remains relatively limited [71]. Therefore, the energy dissipation and waveform distortion caused by geometrical dispersion account for only a small proportion of the overall response, and the axial dominant stress wave still governs the overall plastic deformation process. As a result, the proposed one-dimensional equivalent model can still accurately capture the dominant energy transfer mechanism and overall deformation evolution during ultrasonic impact treatment.

### 5.2 Experimental results for 6061-T6 Aluminum Alloy

#### 5.2.1 Comparison of FEA and equivalent single impact test

**Fig. 10:**
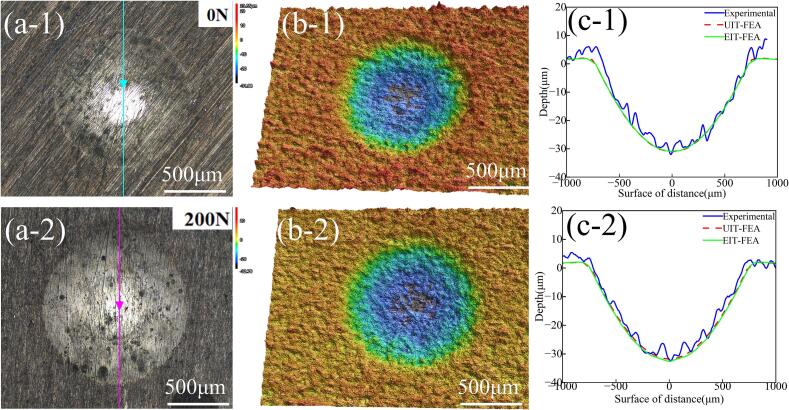
Equivalent impact results for 6061-T6 Aluminum Alloy. (a) Macro profile. (b) 3D morphology. (c) Central cross-section profile.

Experimental results related to equivalent impact are summarized in Fig. 10, where subfigures (a)–(c) represent different combinations of experimental parameters. (a) Under a 5X objective lens, the macro morphology of the impact pit exhibits a distinct spherical crown shaped depression. (b) The high resolution two dimensional projection reveals fine micrometer scale undulations across the surface. (c) The contour profiles extracted along the pit center align closely with the FEA result curves, confirming the geometrical equivalence of the impact pit.

Results from the equivalent impact experiments conducted under static load of 0 N and 200 N are presented in Fig. 10 respectively. There is a distinct ring boundary in each figure, separating the crown shaped impact pit from the surrounding region. The three dimensional topography reveals pronounced surface roughness on the specimen, with noticeable bulging around the pit, suggesting a relatively uniform outward material flow during deformation. The central cross-sectional profiles of pits obtained from the single-cycle ultrasonic impact simulation, the equivalent single impact simulation, as well as the result from the equivalent single impact test, all show good overall consistency.

**Fig. 11:**
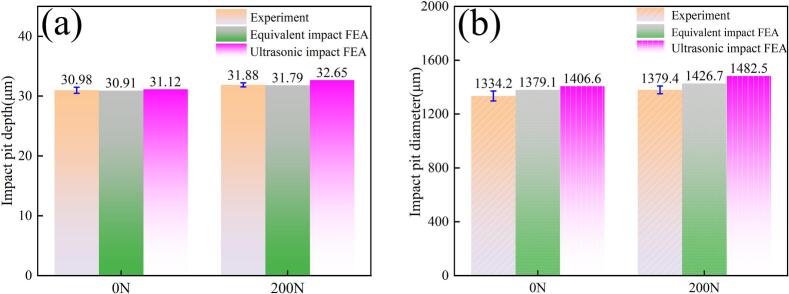
Summary of FEA and test results for single impact on 6061-T6 Aluminum Alloy. (a) Impact pit depth. (b) Impact pit diameter.

Fig. 11 compares the depth and diameter of pits obtained from the single-cycle ultrasonic impact and equivalent single impact FEA with those measured from the equivalent single impact experiments conducted under static load of 0 N and 200 N. The measured pit depths from the equivalent single impact experiments are 30.98 μm and 31.88 μm,with corresponding diameters of 1334.2 μm and 1379.4 μm respectively. The deviations between the equivalent impact simulation and experimental results did not exceed 0.2% for depth and 3.2% for diameter, while those between the single-cycle ultrasonic impact simulation and experimental results did not exceed 2.3% and 6.9% respectively. These results indicate that the simulation model achieves very high predictive accuracy for impact pit depth, with the predicted diameter remaining within an acceptable range.

#### 5.2.2 Comparison of UIT and multiple EIT test

**Fig. 12:**
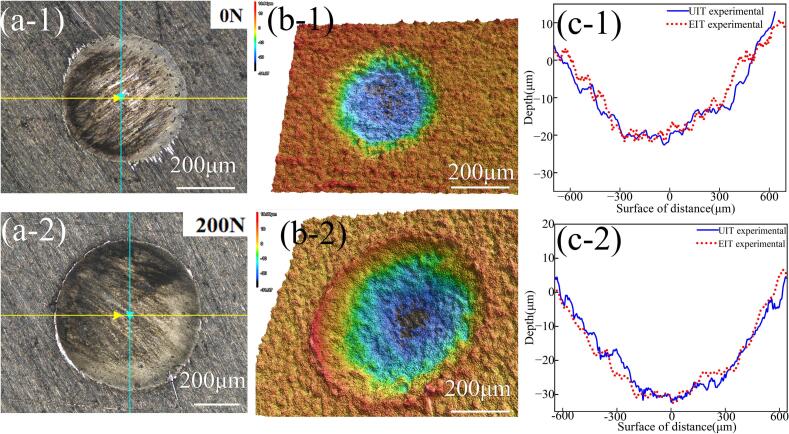
UIT and EIT results for 6061-T6 Aluminum Alloy. (a) Macro profile. (b) Three-dimensional morphology. (c) Central cross-sectional profile.

Fig. 12 compare the central cross-sectional profiles of pit formed by 5 s ultrasonic impact and by 30 equivalent impacts under static load of 0 N and 200 N,Where b is the specific size measurement chart. The pits produced by both processes show excellent consistency, with discrepancies in both depth and diameter being less than 2.9%. Further observations reveal that the 6061-T6 Aluminum Alloy workpiece treated with ultrasonic impact displays superior surface quality compared with that processed by the equivalent impact method. This improvement arises from the repeated squeezing action of the impact head during ultrasonic impact, which induces plastic deformation and progressively flattens surface micro protrusions. Consequently, the surface micro height distribution becomes more uniform, and surface roughness is significantly reduced.

**Fig. 13:**
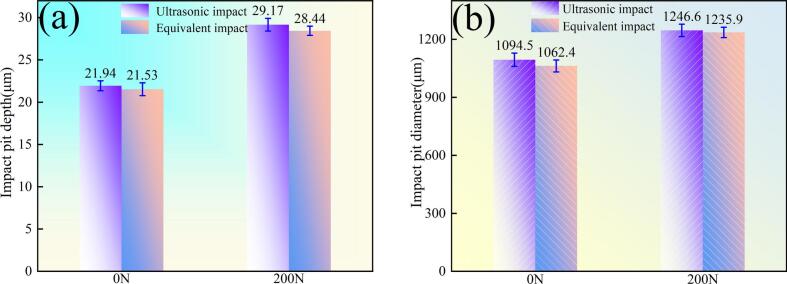
6061-T6 Aluminum Alloy ultrasonic impact and equivalent impact test results. (a) Impact pit depth. (b) Impact pit diameter.

**Fig. 14:**
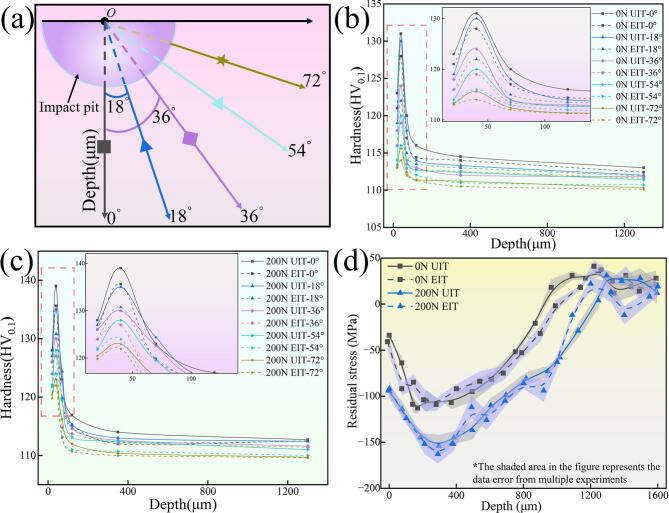
6061-T6 Aluminum Alloy test results. (a).Angle measurement diagram. (b) Hardness 0 N. (c) Hardness 200 N. (d)Data chart of residual stress.

Fig. 13 and Fig. 14(d) present the experimental comparison between ultrasonic impact and 30 equivalent impacts conducted under static load of 0 N and 200 N. The deviations in impact pit depth are 1.8% and 2.5% respectively, the diameter deviations are 2.9% and 0.8%. Under two static load conditions, the maximum RCS amplitude measured by UIT were −113 MPa and −154 MPa respectively, while those measured by EIT were −109 MPa and −163 MPa respectively. The corresponding maximum errors of residual stress were 3.5% and 5.5% respectively. The study found that as the static load increases, the maximum value of RCS and its corresponding depth exhibits a significant deepening trend, indicating that static load can effectively promote the expansion of the residual stress field into deeper regions, thereby forming a thicker strengthened affected layer.

Fig. 14 compares the Vickers hardness test results after ultrasonic impact and 30 equivalent impacts from the perspective of strain hardening. Microhardness gradient measurements were performed along five directions as shown in Fig. 14(a) (0°, 18°, 36°, 54°, 72°) within the impact zone, typical two stage distribution pattern was observed in hardness depth profiles: an initial increase followed by a pronounced decrease phase, subsequently decaying gradually to the matrix hardness level. Although the macroscopic plastic deformation zone formed by the impact extends only tens of micrometres in depth, the hardening influence zone it induces extends far beyond the deformation zone dimensions. Furthermore, the hardening effect's influence range increases with proximity to the impact centre. Under static load conditions of 0 N and 200 N, the hardness distribution curves measured at different angles demonstrate a high degree of consistency between the hardening patterns induced by UIT and EIT. The hardness peaks for both processes occur along the impact normal: UIT exhibits peak hardness values of 131 and 139HV_0.1_ at the two load levels, while EIT yields corresponding values of 128 and 136HV_0.1_ Along each measurement direction, the gradient trends of the hardness distribution curves for both processes largely coincide, with maximum measured deviations not exceeding 5.2% and 4.9% respectively.This experimentally validates that the equivalent impact accurately reproduces the strain hardening mechanism of ultrasonic impact[72],providing a reliable process foundation for actively regulating material surface properties.

#### 5.2.3 Analysis and comparison of UIT and multiple EIT EBSD results

**Fig. 15:**
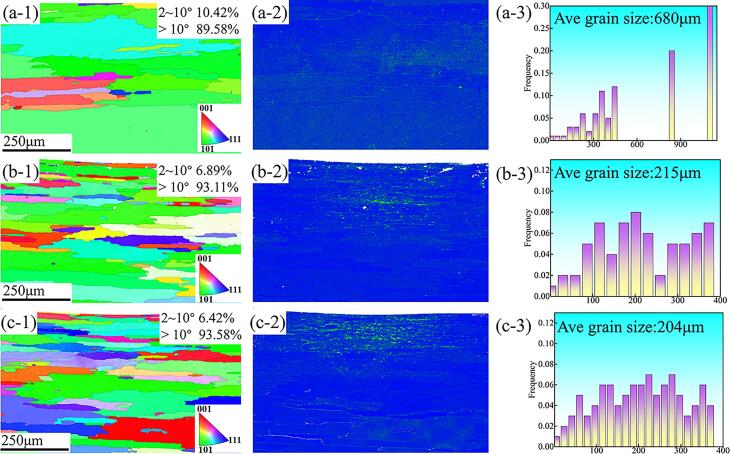
EBSD grain distribution results for impact pits in 6061-T6 Aluminum Alloy. (a) Untreated region. (b) EIT. (c) UIT.

Fig. 15 presents the inverse pole figure (IPF) maps and grain size distribution characteristics of 6061-T6 Aluminum Alloy under different processing conditions. The initial specimen (Fig.15a-1) exhibits a typical extruded microstructure, in which grains are significantly elongated along the processing direction, with an average grain size as large as 680 μm. After the application of EIT (Fig. 15b-1) and UIT (Fig. 15c-1), the coarse grains in the surface layer undergo severe deformation under high strain-rate loading, resulting in a pronounced gradient grain refinement. Quantitative analysis shows that the average grain size decreases sharply from 680 μm to 215 μm after EIT, and further to 204 μm after UIT The two processes not only exhibit highly comparable refinement magnitudes but also display a strong consistency in the grain size distribution bandwidth, which provides strong evidence for the capability of the equivalent impact model to accurately simulate the microstructural deformation process occurring in the surface layer of aluminum alloys.

From the perspective of microscopic strain evolution, the kernel average misorientation (KAM) maps (Fig. 15a-2, b-2, c-2) clearly illustrate the strain field distribution induced by impact loading. In the initial state, the stored energy within the material is relatively low, and the KAM map exhibits a uniformly dark-blue tone, indicating a very low level of lattice distortion within the grains. After the two impact treatments, regions of high strain concentration appear in the surface layer of the specimens. Moreover, the strain-affected depth and gradient distribution characteristics induced by the EIT and the actual UIT are largely consistent. This agreement further demonstrates that the two processes share a highly similar microscopic energy dissipation mechanism.

Quantitative analysis of the Grain Boundary Characteristic Distribution (GBCD) further elucidates the underlying mechanisms of microstructural evolution. In the initial specimen, high-angle grain boundaries (HAGBs, >10°) account for 89.58%, while low-angle grain boundaries (LAGBs, 2°-10°) represent 10.42%. Following the EIT, the proportion of HAGBs surges to 93.11%, and further increases to 93.58% after UIT. This significant rise in the HAGBs fraction under both processes indicates that severe plastic deformation effectively promotes grain refinement and misorientation evolution. The high degree of synchronization in grain boundary evolution between the two methods profoundly reveals that the equivalent impact model can accurately replicate the substructural evolution mechanisms under ultrasonic impact conditions.

### 5.3 Experimental results for 45# steel

#### 5.3.1 Comparison of FEA and equivalent single impact test

**Fig. 16:**
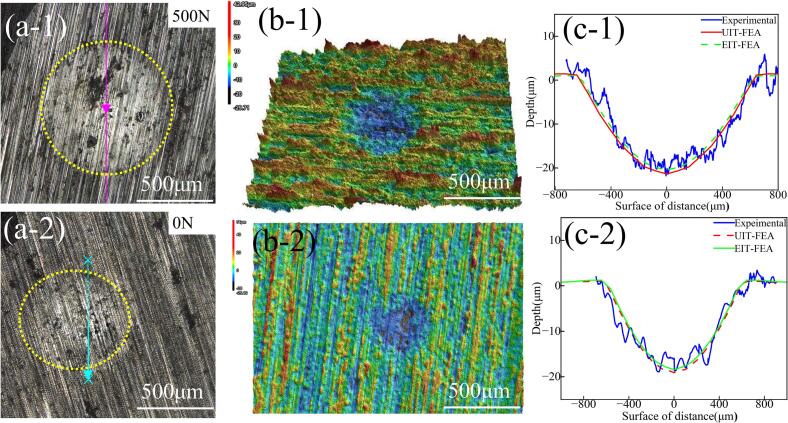
Microstructural results of 45# steel. (a) Macro profile. (b) Three-dimensional morphology. (c) Central cross-sectional profile.

The results of equivalent impact test on 45**#** steel under static load of 0 N and 500 N are shown in Fig. 16. It can be observed that the higher yield strength of 45**#** steel limits the development of plastic deformation, resulting in significantly smaller indentation size compared to the previously discussed 6061-T6 Aluminum Alloy specimens. The surface topography of the specimen exhibits distinct roughness characteristics both before and after impact.

The simulation model demonstrated high predictive accuracy for the indentation depth in 45**#** steel. Although the accuracy in predicting the indentation diameter is slightly lower, it remains within a reasonable range. This reduced accuracy may be attributed to the relatively rough surface of the 45**#** steel specimens. Nevertheless, the overall prediction results exhibit high precision and reliability, confirming that the proposed simulation approach possesses strong robustness and broad applicability across different materials.

It is worth noting that the initial surface roughness has a certain influence on the local contact behavior of high-strength materials during the impact process. When obvious asperities and valleys exist on the specimen surface, the actual contact condition at the initial impact stage deviates from the ideal smooth-contact assumption, thereby inducing local stress concentration, stress-wave scattering, and additional energy dissipation. For high-strength materials such as 45# steel and 1Cr12Ni3Mo2VN, due to their relatively high yield strength, the surface asperities cannot be rapidly plastically flattened during the early stage of impact, making the roughness effect more pronounced.

To further investigate this effect, supplementary single-impact experiments under polished surface conditions were conducted in this study. The results (see Appendix Figs 4–7) show that, as the surface roughness decreases, the agreement between the experimental results and the equivalent model is further improved, indicating that reducing surface roughness contributes to enhancing the prediction accuracy of the model. In addition, the introduction of static pressure can further improve the impact contact condition. Under higher static pressure, a more stable initial contact state is formed between the impact tip and the specimen, and the surface asperities are partially pre-compacted prior to impact, thereby weakening the disturbance of local contact nonlinearity on stress-wave propagation.

**Fig. 17:**
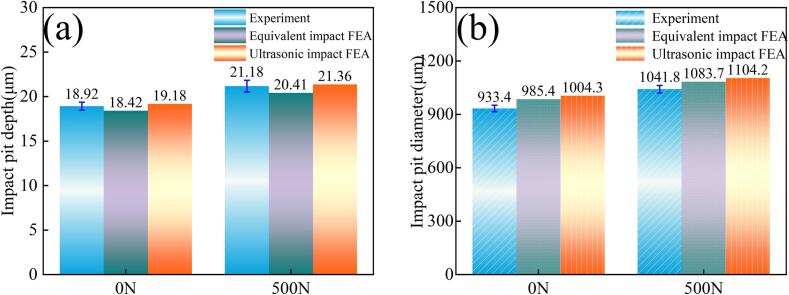
Summary of FEA and experimental results for 45# steel. (a) Impact pit depth. (b) Impact pit diameter.

Fig. 17 compares the indentation depth and diameter obtained from the equivalent impact experiments and FEA under static load of 0 N and 500 N. The experimentally measured indentation depths are 18.92 μm and 21.18 μm, with corresponding diameters of 933.4 μm and 1041.8 μm. In the equivalent impact simulation under static load 0 N, the prediction deviations for depth and diameter are within 2.6% and 5.2%, while in the single-cycle ultrasonic impact simulation, the deviations are within 1.3% and 7%. The overall error distribution remained within an acceptable range.

According to the quantitative analysis presented in Fig. 17, under a static load of 500 N, the measured diameter of the impact pit is 1041 μm, showing an absolute deviation of 42 μm from the EIT-FEA prediction of 1083 μm, corresponding to a relative error of 3.8%,the absolute deviation from the UIT-FEA predicted value of 1104 μm is 63 μm, with a relative error of 5.7%. Meanwhile, the measured maximum indentation depth along the pit’s normal axis is 21.18 μm, with a relative error of 0.8% compared to the FEA predicted value of 21.36 μm for the single-cycle ultrasonic impact simulation. When compared to the FEA prediction value of 20.41 μm for a single equivalent impact, the relative error was 3.6%. Under observation with a 5X objective lens, the 500 N static load induced a pronounced plastic deformation effect in the impact pit of 45**#** steel—the maximum indentation depth increased by 2.26 μm (approximately 12%), and the diameter expanded by 108 μm (11.5%).

The FEA model established based on the coupling mechanism between static and dynamic loads demonstrates high reliability in predicting the characteristics of impact pit. The introduction of static load effectively regulates the material’s stress distribution and plastic flow behavior, thereby significantly amplifying the plastic deformation induced by impact [73]. These results verify the model’s capability to accurately describe the deformation mechanism under coupled loading conditions, providing new insights and theoretical support for the further development and process parameters optimization of ultrasonic impact technology.

#### 5.3.2 Comparison of UIT and multiple EIT test

**Fig. 18:**
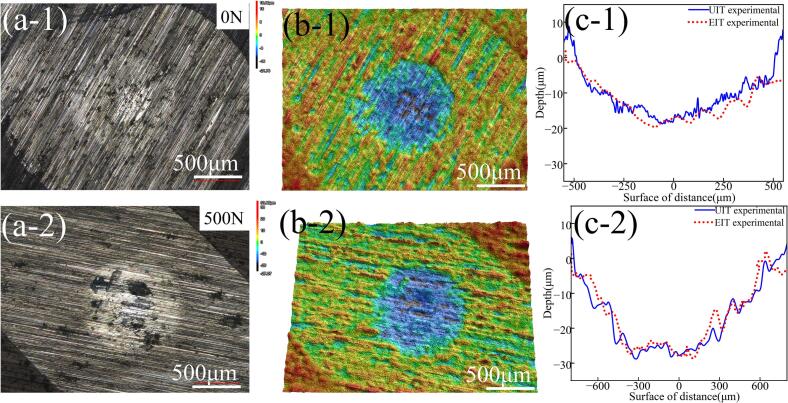
UIT and EIT results for 45# steel. (a) Macro profile. (b) Three dimensional morphology. (c) Central cross-section profile.

**Fig. 19:**
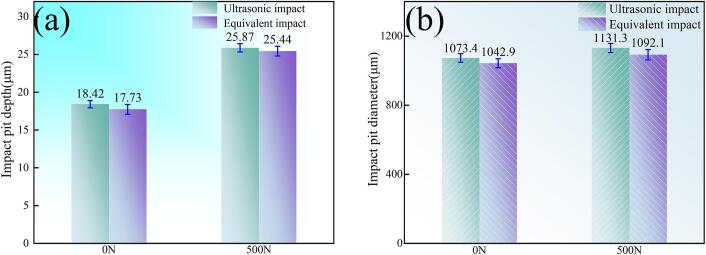
Morphological results of ultrasonic impact and equivalent impact test on 45# steel. (a) Impact pit depth. (b) Impact pit diameter.

To further validate the universality of the proposed method, multiple equivalent impact experiments are performed on 45**#** steel. Fig. 18 compare the morphology of impact pits produced by 30 equivalent impacts and corresponding 5 s ultrasonic impact. The results indicate that, under both static load conditions, the pit morphologies from the equivalent impact closely match those from the ultrasonic impact. As shown in Fig. 19, the depth errors between the equivalent impact pits and ultrasonic impact pits are 3.7% and 1.6% under 0 N and 500 N static load respectively, while the diameter errors are 2.8% and 3.4%. Macro morphology characterization confirms that the impact pits generated by both methods exhibit highly similar geometric features.

Repeated impacts play a positive role in improving the surface roughness, and this phenomenon is observed in both impact processes. Compared with the sharp peak–valley morphology formed after a single impact, the cumulative coverage of impact pits gradually increases during repeated impacts. Under continuous multiple impacts, the initially formed protruding peaks are repeatedly flattened, and the material undergoes cyclic plastic flow and migrates toward the valleys, leading to a gradual smoothing of the originally rough surface.This surface smoothing effect originates from the accumulated plastic deformation induced by repeated impacts. Each impact introduces additional compressive deformation in a localized region, progressively forging and leveling the microscopic asperities. Meanwhile, the material experiences lateral plastic flow under the impact force, filling the adjacent valley regions. As the number of impacts increases, the height difference between surface asperities continuously decreases. Once the impact process reaches a saturation state, the surface roughness gradually stabilizes.

This phenomenon is highly consistent with the observations reported for GH3230 alloy, where the surface gradually becomes smoother with increasing impact duration [74].This agreement indicates that the surface-flattening effect induced by repeated impacts exhibits a certain degree of material universality. These findings therefore provide a theoretical basis for the application of ultrasonic impact treatment in surface finishing processes.

**Fig. 20:**
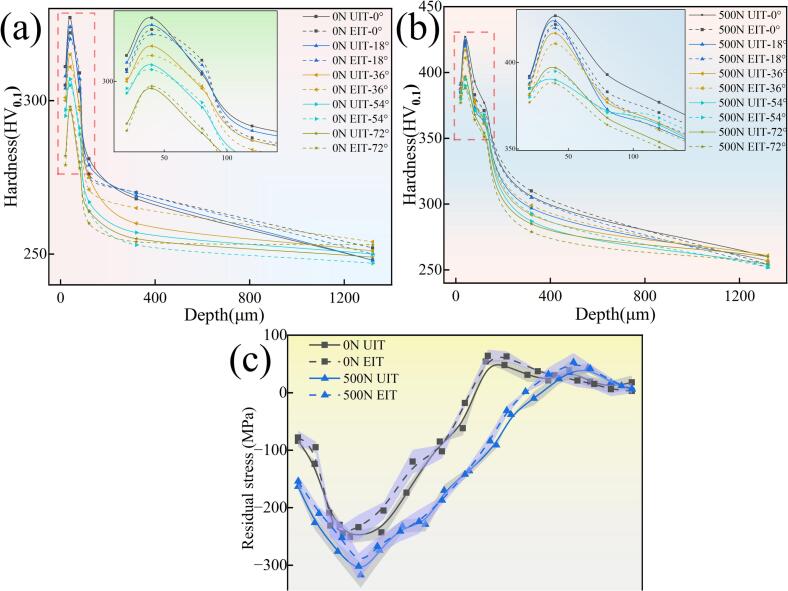
45# steel test results. (a) Hardness 0 N. (b) Hardness 500 N. (c) Residual stress.

Fig. 20(a–b) present the hardness depth profiles of 45**#** steel under different processing conditions. The hardness distribution patterns after the two treatments exhibit a high degree of consistency. For all measurement angles, the hardness peak appears in the subsurface region approximately 40 μm below the impact surface. This characteristic agrees well with the dislocation density distribution revealed by the KAM analysis, where the subsurface region also shows the maximum local misorientation, indicating that this region experienced the most intense plastic deformation and dislocation multiplication, thereby contributing to the highest hardening effect.This phenomenon can be attributed to the severe plastic flow at the surface and the strain gradient effect during the impact process. The hardness peak occurs along the normal direction of impact. Under 0 N static load, the peak hardness values of UIT and EIT are 327 HV_0.1_ and 322 HV_0.1_, with relative errors of 1.3% and 0.9%. Under a 500 N static load, the peak hardness values of both treatments increase correspondingly, while the deviation between the two methods remains within 2%. As the measurement angle increases, the hardness values at the same depth gradually decrease, reflecting the directional dependence of the impact-induced hardening effect, which is consistent with the spatial distribution characteristics of the impact stress field.

A comparison of hardness curves measured at different angles further shows that the maximum deviations between UIT and EIT are less than 1.5% and 2.5%, respectively, and the variation trends of the hardening gradients are essentially identical. The hardening behaviors of the two processes under both 0 N and 500 N static loads demonstrate a high level of agreement, indicating that the equivalent impact model can accurately reproduce the work-hardening behavior induced by ultrasonic impact treatment. This further verifies the universality and reliability of the equivalent impact approach across different material systems.

A further analysis of the hardened layer depth shows that the hardness of specimens treated by both processes remains at a relatively high level within the 0–200 μm surface layer, after which it gradually decreases and approaches the matrix hardness at a depth of approximately 800–1000 μm. This gradient hardening behavior can be explained in terms of the propagation and attenuation mechanisms of stress wave.During the impact process, the stress wave generated at the contact interface propagate into the material in the form of spherical wave. As the propagation depth increases, the wavefront area continuously expands, resulting in a geometric decay of the energy density per unit area. Meanwhile, internal damping and friction within the material continuously dissipate the wave energy, causing the stress amplitude to decrease exponentially with increasing propagation distance.

A direct consequence of stress wave attenuation is that the near surface region experiences sufficiently high stress amplitudes to activate intensive dislocation multiplication and motion, thereby forming a high-density dislocation structure. With increasing depth, however, the stress amplitude gradually falls below the dynamic yield strength of the material, leading to reduced plastic deformation and a lower dislocation density, which is reflected macroscopically as a decrease in hardness.This gradient plastic deformation mechanism governed by stress wave energy dissipation is the fundamental reason why a gradient hardened layer from the surface to the interior is formed after impact treatment. It also provides a theoretical basis for optimizing impact parameters to regulate the depth of the hardened layer.

As shown in Fig. 20(c), the comparative analysis of residual stress along the depth direction in 45**#** steel, two methods demonstrate strong consistency under both 0 N and 500 N static load conditions, with highly aligned distribution curves for each parameter set. A comparison was conducted between the residual stresses induced by UIT and EIT under varying static load conditions. Experimental results indicate that at lower static load, the maximum RCS amplitude introduced by UIT was −251 MPa, while the corresponding maximum RCS amplitude for EIT was −245 MPa, exhibiting an absolute deviation of 9 MPa and a relative error of approximately 2.3%. At higher static load, the maximum RCS induced by UIT increased to −317 MPa, while EIT reached −302 MPa. The absolute deviation was 15 MPa, with a relative error of approximately 4.7%. This data set further confirms that the equivalent impact technique exhibits good consistency with the original ultrasonic impact process regarding the introduction level of RCS, a key mechanical property indicator. The application of static load increases the peak residual stress by approximately 70 MPa.

This phenomenon can be further interpreted in terms of the propagation and attenuation mechanisms of stress wave. The energy dissipation from the surface to the interior governs the gradient distribution of both plastic deformation and residual stress amplitude along the depth direction. In the near surface region, the concentration of stress wave energy induces severe plastic deformation, which results in the formation of high-amplitude residual compressive stresses after unloading. As the depth increases, the strain energy driven by the stress-wave amplitude gradually decreases. Once it falls below the dynamic yield strength of the material, plastic deformation becomes weaker and the residual stress correspondingly diminishes.

Based on the analysis of coupling mechanism between static and dynamic loads, the FEA model demonstrates high reliability in predicting key features of impact pit. The introduction of static load improves significantly modifies the material’s stress state and plastic flow behavior, thereby amplifying the impact induced deformation. The findings demonstrate that the proposed equivalent impact method effectively simulates the plastic deformation mechanisms of both low-strength 6061-T6 Aluminum Alloy and high-strength 45**#** steel, confirming its robust applicability. This work provides a solid mechanical foundation for understanding and optimizing ultrasonic impact.

#### 5.3.3 Analysis and comparison of UIT and multiple EIT EBSD results

**Fig. 21:**
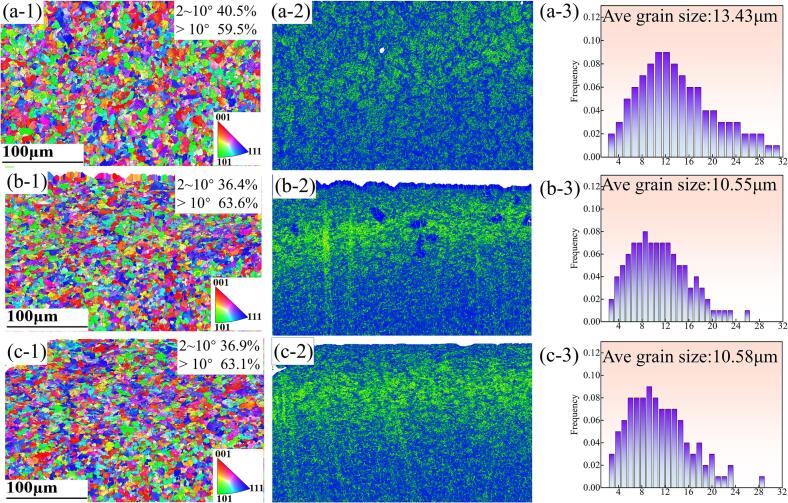
EBSD grain distribution results for impact pit in 45#steel. (a) Untreated region. (b) EIT. (c) UIT.

Fig. 21 systematically presents the surface microstructural characteristics of 45**#** steel in the initial state and after different impact treatments. As shown in Fig. 21(a), the original specimen exhibits a typical quenched and tempered microstructure, with an average grain size of approximately 13.43 μm. The grain boundaries are clearly defined, and the internal crystal orientation appears relatively uniform. In contrast, after EIT Fig. 21b and UIT Fig. 21c, the surface layer of the specimens undergoes pronounced strain-induced refinement. Under the action of high-frequency and high-intensity loading, the original coarse grains are significantly refined, resulting in the formation of a distinct gradient nano–microstructure near the surface.Quantitative analysis shows that the average grain size decreases sharply to 10.55 μm after EIT, while it reaches 10.58 μm after UIT. The difference in grain refinement between the two treatments is extremely small. This highly consistent refinement behavior strongly demonstrates the accuracy of the equivalent impact model in reproducing the plastic deformation response induced by actual ultrasonic impact treatment.

Further analysis based on KAM and dislocation density reveals the consistency of the strengthening mechanisms. The initial specimen exhibits a dislocation density of 5.9 × 10^14^ m^−2^. After EIT and UIT, the dislocation densities increase significantly to 8.2 × 10^14^ m^−2^ and 8.9 × 10^14^ m^−2^.The two treatments show a high degree of consistency in both numerical magnitude and distribution characteristics.With the rapid multiplication of dislocations and the progressive refinement of grains, the fraction of low-angle grain boundaries (LAGBs) in the treated specimens increases to 63.6% and 63.1%, respectively, representing an increase of approximately 23% compared with the initial state. This highly consistent grain boundary distribution and strain field characteristic fundamentally demonstrates that the equivalent impact model is capable of reproducing the macroscopic grain refinement behavior observed in ultrasonic impact treatment.

During the impact process, the input energy is transmitted into the material in the form of stress waves, which gradually dissipate with increasing depth. This energy dissipation governs the gradient variation of plastic deformation along the depth direction. By accurately reproducing this energy input and dissipation process, the equivalent impact model enables a comprehensive simulation of the microscopic strengthening mechanisms induced by ultrasonic impact treatment.

A comprehensive analysis integrating IPF maps, KAM distributions, and grain boundary characteristics leads to the conclusion that the equivalent impact model established in this study exhibits a high degree of consistency with the actual UITt in terms of microstructural evolution. The model is capable of accurately reproducing the grain refinement process and strain field distribution characteristics under severe plastic deformation. These results demonstrate that the proposed approach provides a reliable experimental simulation platform for the optimization of UIT parameters and for the prediction of micro-scale mechanical behavior.

## 6 Conclusion

(1) This study proposes an analytical method for quantifying dynamic ultrasonic impact loads based on stress wave propagation theory and the principle of energy equivalence, which can effectively reproduce the impact process within each cycle. An elastoplastic finite element model was developed to simulate the interaction between impact stress wave and material response, enabling the quantitative prediction of single impact dynamic load. Furthermore, the model was validated using a self-developed equivalent ultrasonic impact test apparatus for two engineering materials with significantly different strength —— 6061-T6 Aluminum Alloy and 45**#** steel. A comprehensive multi-dimensional evaluation, encompassing FEA results, macroscopic morphologies, forming profiles, grain gradients, and strain hardening effects, indicates that this equivalent approach is accurate and feasible. This approach offers a novel perspective for elucidating the evolution of material properties during ultrasonic impact processe.
(2) The feasibility of simulating ultrasonic periodic impact by exciting stress wave with an impact rod is confirmed. By introducing a rubber pad to modify the waveform, the stress wave generated by the equivalent impact closely matches that of ultrasonic impact. Experimental measurements confirm that the equivalent impact stress waveform closely matches the theoretical ultrasonic impact waveform, with deviations of amplitude and pulse width < 4.4% and 1 μs respectively, thereby validating the accuracy of the model derivation Single equivalent impact and single-cycle ultrasonic impact exhibit a high degree of consistency. Multi-dimensional validation confirms the high feasibility of a single equivalent impact, with three dimensional morphological analysis showing an impact pit diameter deviation < 7% and depth error < 3.6%.
(3) For both 6061-T6 Aluminum Alloy and 45**#** steel, the accuracy of the equivalent impact method in simulating multiple impacts surpasses that of single impact, as key interference factors such as surface roughness are eliminated. The achieved accuracy confirms the method’s robust cross-material applicability and validates its effectiveness and engineering universality. The equivalent model demonstrated excellent consistency in simulating a 5 s ultrasonic impact, accurately reproducing the effects of ultrasonic impact. The two specimens exhibit high agreement in key three dimensional morphological parameters, with average diameter deviation < 3.4%, depth error < 3.7%, hardness difference < 5.2%, and maximum residual stress variation ≤ 5.5%. These results are attributed to the equivalent model's effective accurate capture of the cumulative effects and dynamic response of ultrasonic impact, establishing a reliable research benchmark for ultrasonic impact parameters optimization, also provides a new method for studying the modification mechanisms of ultrasonic impact.
(4) In ultrasonic impact processing, under fixed dynamic load energy input conditions, the higher the material's initial strength, the more pronounced the enhancement effect of static load on surface strengthening. Analysis of multiple sets of hardness measurement data reveals that stress waves generated by dynamic load during ultrasonic impact exhibit maximum energy density in the direction perpendicular to the workpiece surface. This results in significantly superior strengthening effects in the normal direction compared to other orientations.The present study provides an effective research tool to reveal the deformation and strengthening mechanisms during ultrasonic impact, paving the way for parameter optimization(of ultrasonic impact treatment).

In summary, the equivalent model and experimental framework developed in this study demonstrate significant scientific and practical value. By accurately decoupling the high-frequency characteristics of ultrasonic impact, this approach provides a novel research paradigm for exploring high-frequency dynamics. Its exceptional adaptability to both single and multiple impact scenarios ensures a precise correlation with the resulting mechanical properties. Looking forward, this work enables the investigation of the evolution laws governing impact parameters and mechanical performance across varying impact cycles. Furthermore, through diverse characterization techniques, the internal structural evolution under cumulative impacts can be systematically observed. The authors firmly believe that this methodology provides a fundamental equivalence for the ultrasonic impact process, establishing a scientific and universal theoretical model for understanding material behavior under complex impact dynamics. It also offers a research approach for the low-frequency visualization of other ultrasonic-based processes.

## 7 Limitations and future works

This study is conducted based on the one-dimensional stress wave propagation theory. However, it should be noted that the strict one-dimensional stress wave theory is generally applicable to slender rod structures, whereas the actual ultrasonic impact system in the present work possesses finite lateral dimensions. Therefore, certain geometrical dispersion effects and transverse vibration influences inevitably exist. Nevertheless, due to the relatively short stress-wave propagation distance in the present impact system, no obvious long-distance dispersion accumulation occurs during propagation. As a result, the waveform distortion and energy dissipation caused by geometrical dispersion remain relatively limited. Under such conditions, the axial dominant stress wave still governs the overall deformation behavior, and thus the stress-wave propagation process in this study is approximately treated as one-dimensional.

Despite this, the proposed one-dimensional equivalent model still has certain limitations. First, the one-dimensional theory cannot fully describe the higher-order vibration mode coupling induced by transverse inertia in finite-sized structures; therefore, some deviations remain in predicting local high-frequency oscillation characteristics. Second, the theoretical model adopts an ideal continuous-contact assumption and does not fully account for rough surface contact, local sliding, and nonlinear contact stiffness variations at the actual impact interface. In addition, for the high-frequency attenuation behavior observed in experimental stress waves, the one-dimensional theory does not consider the influences of material damping, interface scattering, or the bandwidth limitations of the measurement system. Consequently, certain local differences still exist between the experimental waveforms and theoretical predictions. Future work will further incorporate two-dimensional/three-dimensional wave propagation and geometrical dispersion effects to improve the model’s capability in describing local high-frequency responses.

Through comprehensive multi-perspective validation, this study has demonstrated the accuracy of the proposed equivalent method. However, due to space limitations, specific practical application cases of the method are not presented in this paper. In future work, the developed equivalent impact experimental platform will be utilized to systematically investigate the performance evolution behavior of ultrasonic impact treatment under different impact cycles, as well as to explore the optimal processing parameters for minimizing surface roughness. Furthermore, the platform can also be used to study the influence of process parameters under different coverage conditions.

It should be noted that the current fixture system of the platform is only suitable for plate specimens and cannot effectively clamp cylindrical rod specimens. Therefore, systematic studies on rod-type specimens cannot yet be conducted. In future work, new fixture systems will be designed for cylindrical specimens to further improve the experimental platform.

## CRediT authorship contribution statement

**Qingshan Jiang:** Writing – review & editing, Supervision, Project administration, Methodology, Funding acquisition, Conceptualization. **Tong Ran:** Writing – review & editing, Writing – original draft, Visualization, Supervision, Formal analysis, Data curation, Conceptualization. **Yongqing Lai:** Writing – review & editing, Visualization, Software, Methodology, Data curation. **Peihan Lin:** Writing – review & editing, Visualization, Validation, Investigation. **Pengbo Qian:** Methodology, Formal analysis, Conceptualization. **Zhilong Xu:** Visualization, Validation. **Fei Sun:** Validation, Investigation.

## Declaration of competing interest

The authors declare that they have no known competing financial interests or personal relationships that could have appeared to influence the work reported in this paper.

## References

[1] Yuan Y. (2024). Review on numerical simulation of ultrasonic impact treatment (UIT): present situation and prospect. J. Mater. Res. Technol..

[2] Mahtabi M. (2026). Cryogenic ultrasonic fatigue: mechanisms, advancements, and insights. Cryogenics.

[3] Liu R. (2021). Application of ultrasonic nanocrystal surface modification (UNSM) technique for surface strengthening of titanium and titanium alloys: a mini review. J. Mater. Res. Technol..

[4] Zhou Y. (2025). Study on the microstructure, wear and corrosion resistance of CoCrFeNiMn high-entropy alloy coating via ultrasonic impact treatment. Surf. Coat. Technol..

[5] Lu Y. (2025). Investigation on the microstructure variation of LZ91 Mg-Li alloy by ultrasonic nanocrystal surface modification. J. Alloy. Compd..

[6] Li L. (2016). Influence of multiple ultrasonic impact treatments on surface roughness and wear performance of SUS301 steel. Surf. Coat. Technol..

[7] Zhang Y. (2025). Exploring the strengthening mechanisms of additive manufactured metals treated by ultrasonic nanocrystal surface modification. Int. J. Fatigue.

[8] Ye H. (2019). Effect of ultrasonic surface rolling process on mechanical properties and corrosion resistance of AZ31B Mg alloy. Surf. Coat. Technol..

[9] Cao X.J., Pyoun Y.S., Murakami R. (2010). Fatigue properties of a S45C steel subjected to ultrasonic nanocrystal surface modification. Appl. Surf. Sci..

[10] Zhao J., Dong Y., Ye C. (2021). Optimization of residual stresses generated by ultrasonic nanocrystalline surface modification through analytical modeling and data-driven prediction. Int. J. Mech. Sci..

[11] Wang G. (2024). Gradient residual stress evolution and its influence on fatigue life under combined carburising heat treatment and ultrasonic surface rolling process. Eng. Fract. Mech..

[12] Han S. (2026). Refinement effect of ultrasonic impact treatment on fatigue life of welded joints: a quantitative analysis based on crystal plasticity. Eng. Fract. Mech..

[13] Zhang Q. (2025). Plastic deformation behavior and strengthening mechanism induced by ultrasonic impact treatment in CoCrFeNiB0.15 high entropy alloy coatings. J. Manuf. Process..

[14] Zhang K. (2025). Improving fatigue performance of thin-walled components via synchronous double-sided ultrasonic surface rolling process. J. Mater. Process. Technol..

[15] Xiang J. (2025). Analytical prediction of surface morphology and residual stress induced by milling and ultrasonic surface rolling. CIRP J. Manuf. Sci. Technol..

[16] Lin Q. (2022). A CFD-FEM numerical study on shot peening. Int. J. Mech. Sci..

[17] Unal O. (2022). Effects of static load on microstructural and mechanical performance of AISI 1050 medium carbon steel subjected to ultrasonic nanocrystal surface modification. Mater. Sci. Eng. A.

[18] Zhang K. (2024). Effect of high-frequency dynamic characteristics in the ultrasonic surface rolling process on the surface properties. J. Mater. Process. Technol..

[19] Zhang M. (2019). Investigation into contributions of static and dynamic loads to compressive residual stress fields caused by ultrasonic surface rolling. Int. J. Mech. Sci..

[20] Guo C. (2015). Numerical analysis of the residual stress in ultrasonic impact treatment process with single-impact and two-impact models. Appl. Surf. Sci..

[21] Seok T. (2023). New displacement-based finite element analysis method for predicting the surface residual stress generated by ultrasonic nanocrystal surface modification. Eur. J. Mech. A. Solids.

[22] Meng Y. (2020). Tribological properties of textured surfaces fabricated on AISI 1045 steels by ultrasonic surface rolling under dry reciprocating sliding. Wear.

[23] Wang N. (2025). Parameter optimization of ultrasonic impact for deformation control based on dual Information Neural Network. J. Manuf. Process..

[24] Wang S. (2025). Effect of ultrasonic surface rolling process by disparate static pressure on a carburized layer of 17CrNiMo6 steel. Mater. Chem. Phys..

[25] Liu Z. (2025). Microstructure and performance evolution of 18CrNiMo7–6 alloy steel under ultrasonic rolling and sliding wear properties. Mater Charact.

[26] Wang H. (2025). Ultrasonic rolling of 42CrMo Steel: Simulation-based process optimization for high-efficiency and sustainable manufacturing. Ultrason. Sonochem..

[27] Pang Z. (2022). Effect of spindle speed during ultrasonic rolling on surface integrity and fatigue performance of Ti6Al4V alloy. Int. J. Fatigue.

[28] Li G. (2016). Effects of the different frequencies and loads of ultrasonic surface rolling on surface mechanical properties and fretting wear resistance of HIP Ti–6Al–4V alloy. Appl. Surf. Sci..

[29] Lu K. (2021). Experimental investigation of the effects of vibration parameters on ultrasonic vibration-assisted tip-based nanofabrication. Int. J. Mech. Sci..

[30] Zhou W. (2024). The impact of frequency and power on the ultrasonic purification of aluminum alloy. Ultrason. Sonochem..

[31] Ma Y. (2025). Gradient nanostructure and tribological properties of M50 bearing steel treated by ultrasonic surface rolling. Tribol. Int..

[32] Huang P. (2023). Effect of ultrasonic rolling on surface integrity, machining accuracy, and tribological performance of bearing steels under different process schemes. CIRP J. Manuf. Sci. Technol..

[33] Fan K. (2024). Enhanced fretting fatigue strength of TC11 titanium alloy using laser-assisted ultrasonic surface rolling process. Tribol. Int..

[34] Wu J. (2022). Effect of textures fabricated by ultrasonic surface rolling on dry friction and wear properties of GCr15 steel. J. Manuf. Process..

[35] Yao C., Chen J., Tan L. (2024). Experimental investigation on surface integrity and fatigue performance of Ti60 alloy under ultrasonic impact treatment. Eng. Fail. Anal..

[36] Shen L., Liu Y. (2022). Thermo-mechanical coupling shock waves propagation with phase transition under impact loading. Int. J. Impact Eng.

[37] Alotta G., Russillo A.F., Failla G. (2025). Elastic wave propagation in periodic stress-driven nonlocal Timoshenko beams. Int. J. Solids Struct..

[38] Chen L. (2025). Phase geometric propagation model of spherical projectile impacting thin plate based on shock wave propagation. Def. Technol..

[39] Fu J. (2025). The parameter mapping of power ultrasonic transducer model. Ultrasonics.

[40] Jia C. (2026). Design and performance of a novel mode-selectable piezoelectric ultrasonic transducer with controllable output trajectory. Ultrasonics.

[41] Hong Y. (2024). Characterization of microscopic residual stresses: a review. Eng. Fract. Mech..

[42] Zhou C. (2021). Numerical study of the ultrasonic impact on additive manufactured parts. Int. J. Mech. Sci..

[43] Yuan K., Sumi Y. (2016). Simulation of residual stress and fatigue strength of welded joints under the effects of ultrasonic impact treatment (UIT). Int. J. Fatigue.

[44] Zhai J. (2026). Determination of the density in the linear elastic wave equation. J. Differential Equations.

[45] Li Z. (2021). Stress measurement for steel slender waveguides based on the nonlinear relation between guided wave group velocity and stress. Measurement.

[46] Giurgiutiu, V., Chapter 5 - Elastic Waves, in Structural Health Monitoring with Piezoelectric Wafer Active Sensors (Second Edition), V. Giurgiutiu, V. Giurgiutiu^Editors. 2014, Academic Press: Oxford. p. 199-292.

[47] WANG, L., CHAPTER 6 - One-Dimensional Visco-Elastic Waves and Elastic-Visco-Plastic Waves, in Foundations of Stress Waves, L. WANG, L. WANG^Editors. 2007, Elsevier: Oxford. p. 219-264.

[48] Churilov S. (2026). Searching for traveling wave solutions in inhomogeneous moving media by factorizing the wave equation. Wave Motion.

[49] Chen H., Shi B., Guo H. (2026). Numerical analysis and experimental research on the impact characteristics of hydraulic impact hammers based on stress wave theory. Results Eng..

[50] Yang Y. (2019). Characteristics of buried structures in northern Longmenshan mountains and its significance to oil and gas exploration in the Sichuan Basin. Nat. Gas Ind. B.

[51] Kumar D., Ruan D., Khaderi S.N. (2025). Analytical and numerical models to predict the shape of incident pulse in split-Hopkinson bar experiments. Int. J. Impact Eng.

[52] Bagher Shemirani A., Naghdabadi R., Ashrafi M.J. (2016). Experimental and numerical study on choosing proper pulse shapers for testing concrete specimens by split Hopkinson pressure bar apparatus. Constr. Build. Mater..

[53] El Hamoui A.K.M., Hantouche E.G. (2026). Thermal creep behavior in structural steel: explicit vs. implicit approach. Fire Saf. J..

[54] Aravas N., Xenos S. (2023). “Implicit” vs “Explicit” gradient plasticity models: do they always remove mesh dependence in softening materials?. Int. J. Solids Struct..

[55] Song H. (2025). Non-linear analysis of composite structure subjected to impact load based on Johnson-Cook constitutive model. Int. J. Non Linear Mech..

[56] Kuang K. (2025). Optimization of the Johnson–Cook constitutive model for HRB400 steel under high strain rate and high temperature conditions with multi-condition validation. Structures.

[57] Li S. (2021). Optimization of milling aluminum alloy 6061-T6 using modified Johnson-Cook model. Simul. Model. Pract. Theory.

[58] Khan F. (2025). Process parameter optimization and bonding mechanism in dissimilar S45C/A6061 joints via novel sacrificing-sheet linear friction welding. J. Adv. Join. Process..

[59] Gao D. (2021). Critical distance model for the fatigue life analysis under low-velocity impacts of notched specimens. Int. J. Fatigue.

[60] Zhu J. (2026). Recent development in numerical simulation of ultrasonic process enhancement. Chem. Eng. Sci..

[61] Jie Z. (2024). Residual stress distribution and relaxation in U-rib full penetration welds by ultrasonic impact treatment. Structures.

[62] Chen H.Y. (2026). A dual-triple combination characteristic line method for stress wave propagation across the Intact-Defected-Intact (IDI) composite strata. Wave Motion.

[63] Yang H., Li Y., Zhou F. (2022). Stress waves generated in a Rayleigh-Love rod due to impacts. Int. J. Impact Eng.

[64] Lin S., Guo H., Xu J. (2018). Actively adjustable step-type ultrasonic horns in longitudinal vibration. J. Sound Vib..

[65] Li Q. (2021). Strain gauge experimental study on mode I rock fracture characteristics under impact loading. Eng. Fract. Mech..

[66] Ling S. (2026). High-sensitivity electrically isolated differential strain gauge with temperature compensation for precise force measurements. Sens. Actuators, A.

[67] Guo J. (2025). Comprehensive response analysis of stress wave propagation and energy dissipation of a cemented tailings backfill based on the relative dissipation factor. Green and Smart Min. Eng..

[68] Shodja H.M. (2015). Scattering of an anti-plane shear wave by an embedded cylindrical micro-/nano-fiber within couple stress theory with micro inertia. Int. J. Solids Struct..

[69] Shi Z. (2025). The viscoelastic stress wave propagation model based on fractional derivative constitutive. Int. J. Impact Eng.

[70] Rigby S.E., Barr A.D., Clayton M. (2018). A review of Pochhammer–Chree dispersion in the Hopkinson bar. Proc. Inst. Civ. Eng. – Eng. Comput. Mech..

[71] Dong X., Huang X. (2026). Wave dispersion analysis in functionally graded rods: an analytical approach with variable-coefficient equation and Laplace inversion. Int. J. Impact Eng.

[72] Zhou C. (2020). A calculation model to predict the impact stress field and depth of plastic deformation zone of additive manufactured parts in the process of ultrasonic impact treatment. J. Mater. Process. Technol..

[73] Akbar M., Kobayashi S. (2025). Investigating the interchangeability of low-velocity impact and quasi-static indentation Tests: Effects on the residual strength of hybrid aluminum/CFRP laminates. Compos. Sci. Technol..

[74] Chen L. (2025). Effects of ultrasonic shot peening process on the microstructure and mechanical properties of nickel-based superalloys formed by selective laser melting. J. Mater. Process. Technol..

