# Supplementary material for: Energy transfer mechanism in ultrasonic impact and its single-cycle equivalent experimental methodology
Source: Ultrason Sonochem. 2026 Jun 5;130:107910. doi: 10.1016/j.ultsonch.2026.107910 (PMC13263766; doi:10.1016/j.ultsonch.2026.107910)
Supplement: Supplementary Data 1 [file mmc1.docx]

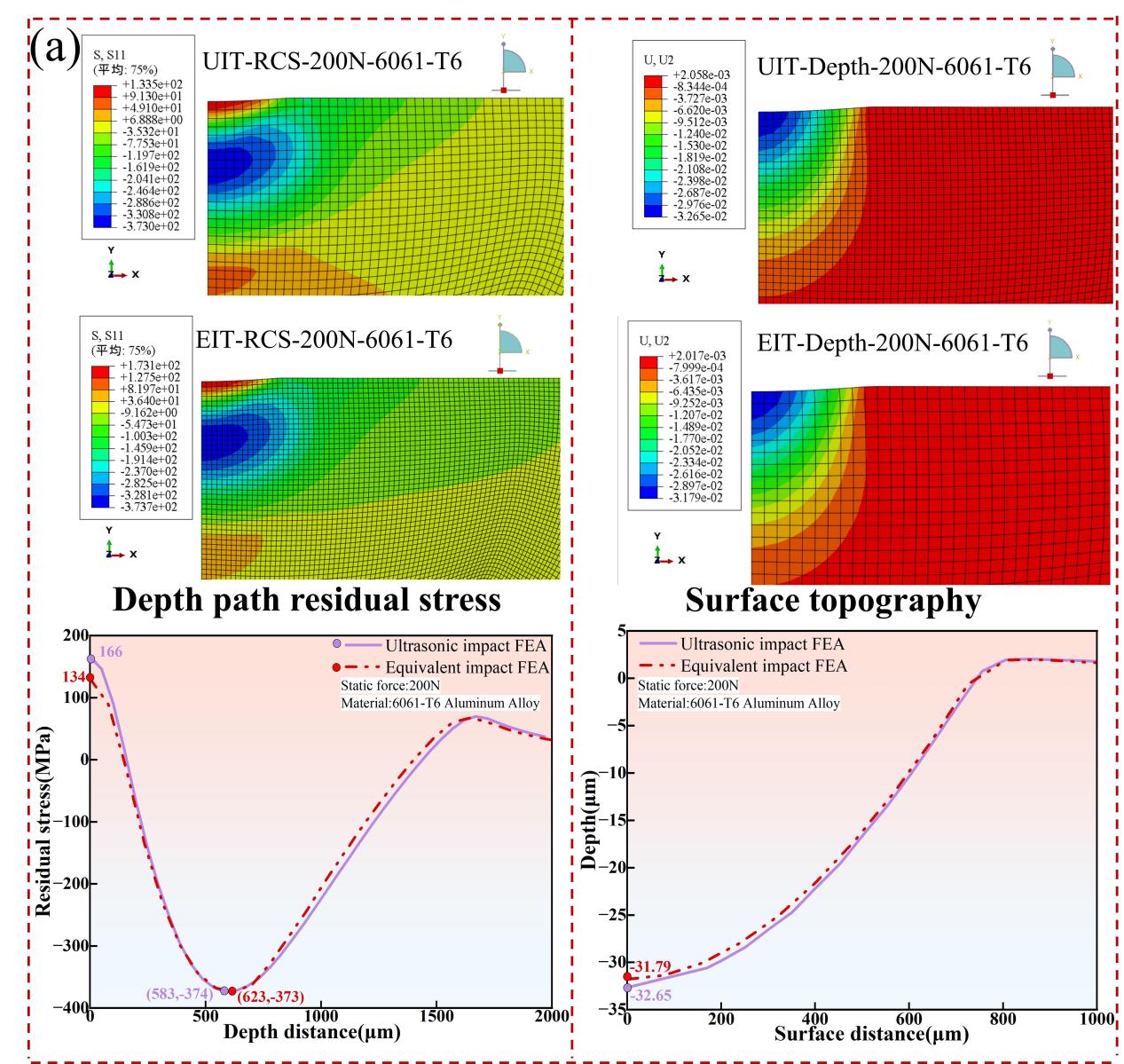


(a) Residual stress contour map. (b) Deformation contour map.

**Fig. 1.** FEA results for EIT and single-cycle UIT on 6061-T6 Aluminum Alloy (200 N).


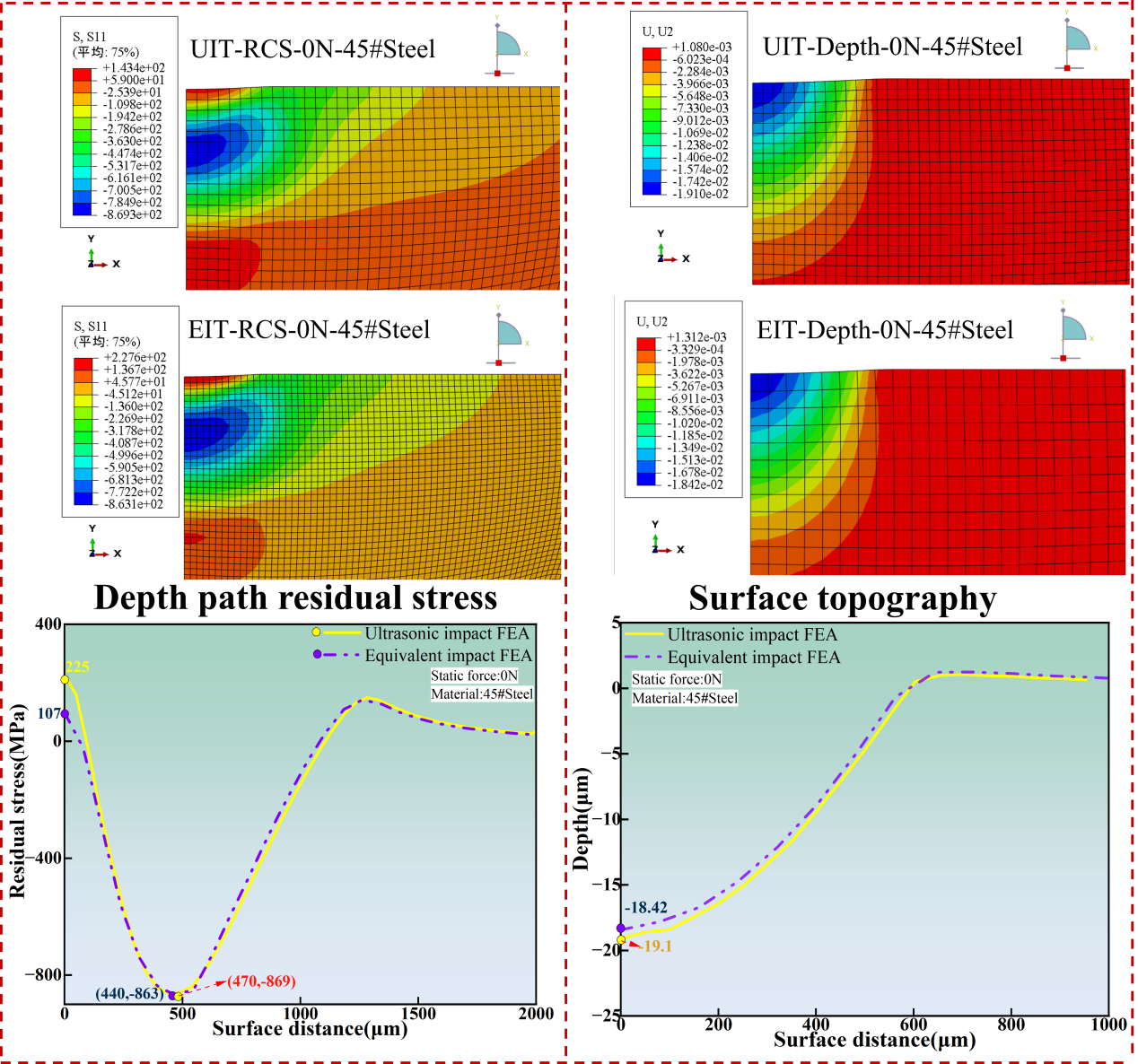


(a) Residual stress contour map. (b) Deformation contour map.

**Fig. 2.** FEA results for EIT and single-cycle UIT on 45**#**Steel (0 N).


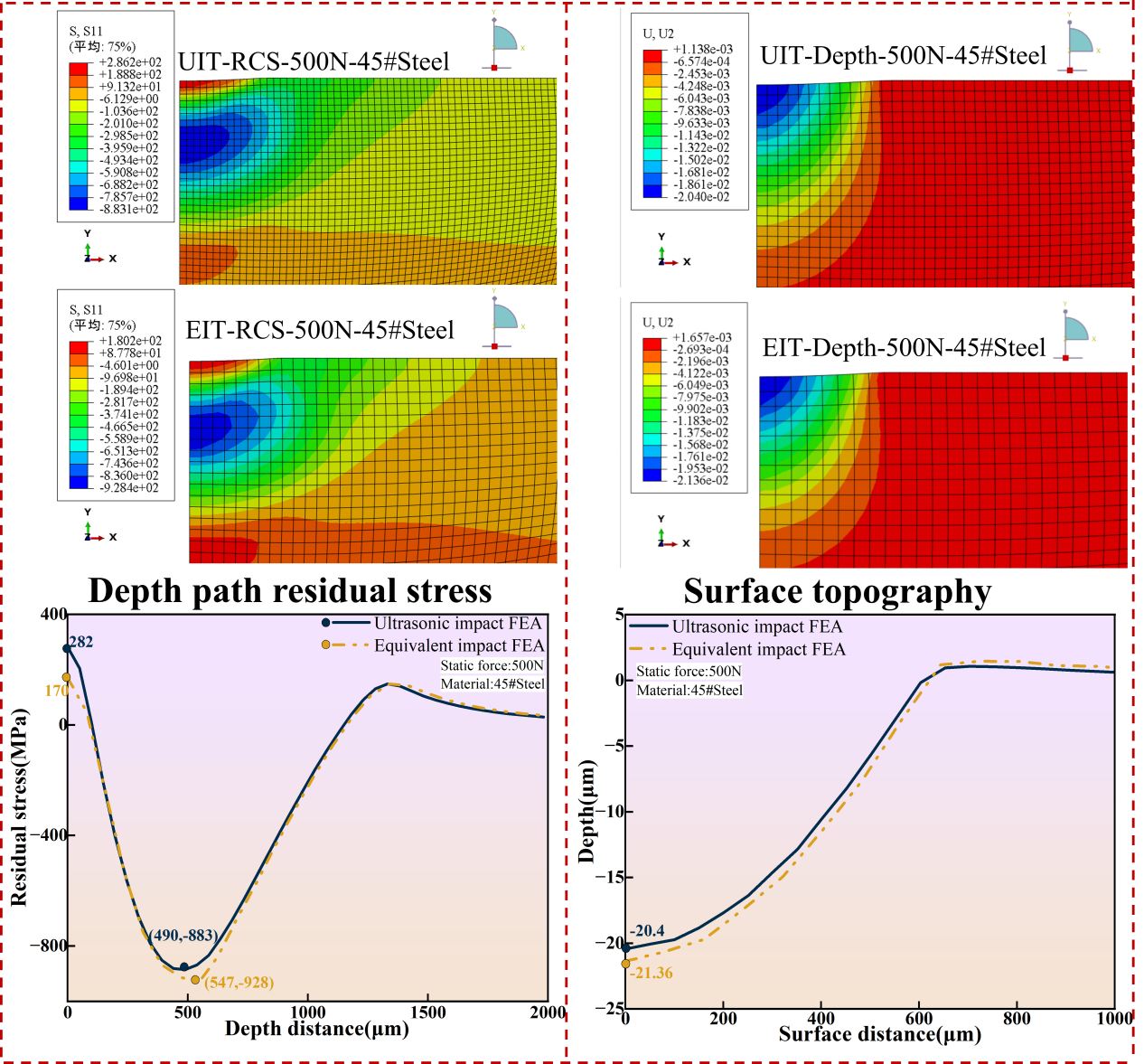


(a) Residual strecontour map. (b) Deformation contour map.

**Fig. 3.** FEA results for EIss T and single-cycle UIT on 45**#**Steel (500 N).


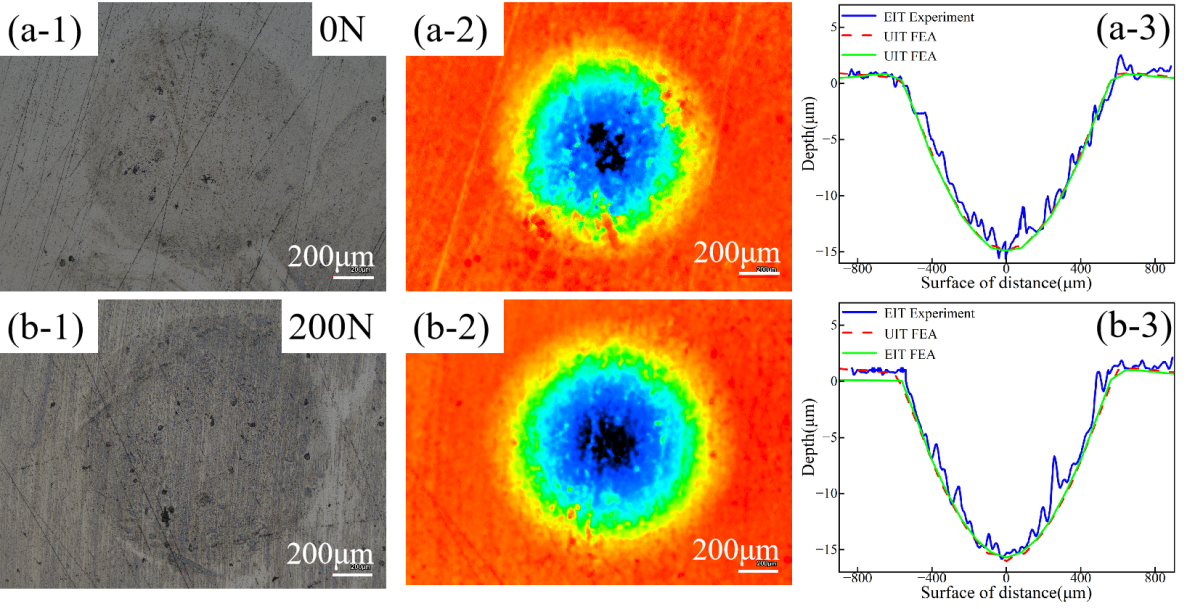


(a) Macro profile. (b) Three-dimensional morphology. (c) Central cross-sectional profile.

**Fig. 4.** Microstructural results of 1Cr12NiMo2VN.


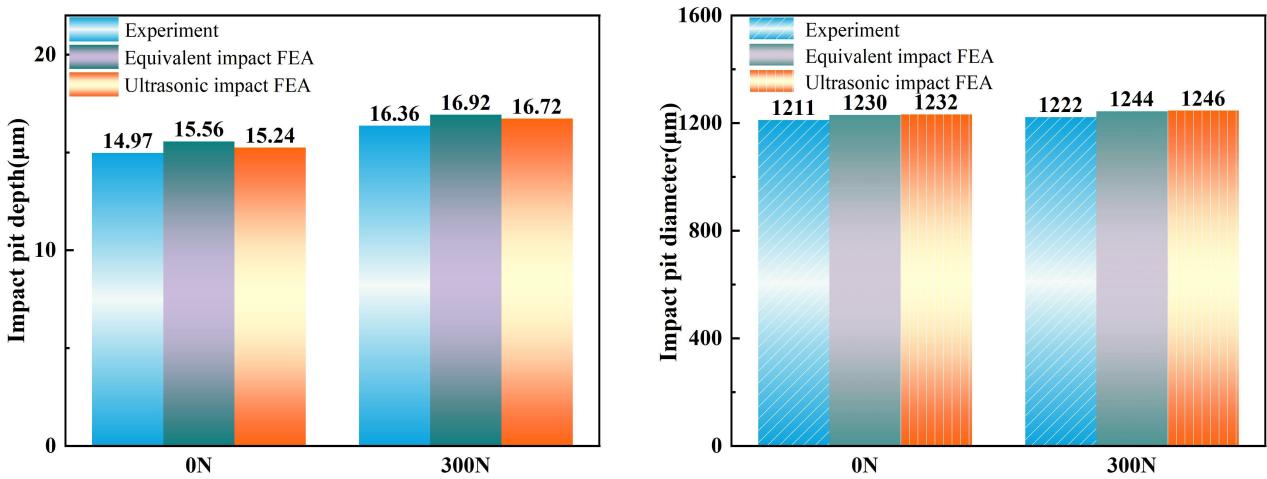


**Fig. 5.** Summary of FEA and experimental results for 1Cr12NiMo2VN.


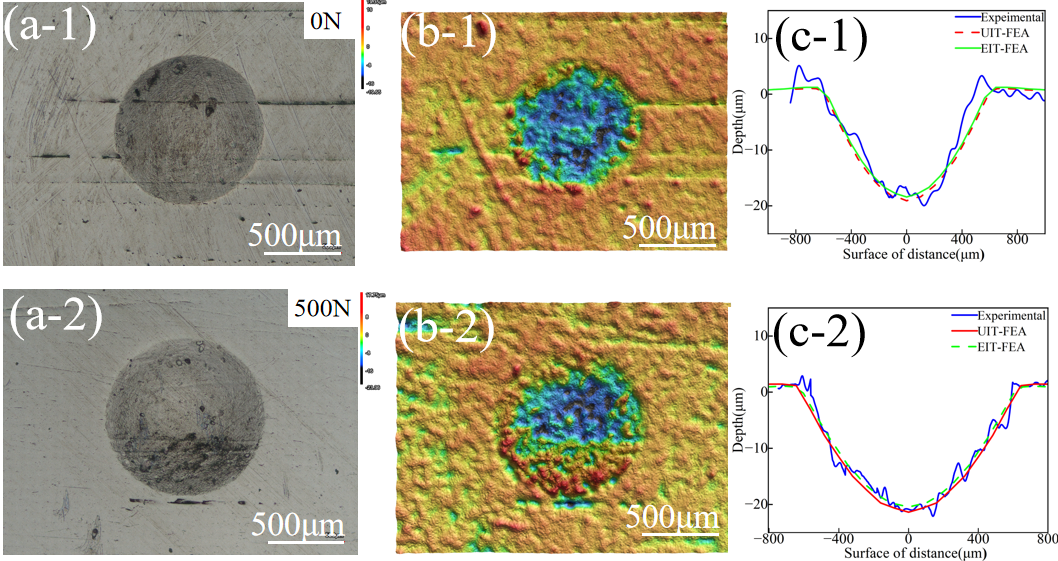


(a) Macro profile. (b) Three-dimensional morphology. (c) Central cross-sectional profile.

**Fig. 6.** Microstructural results of 45# steel.


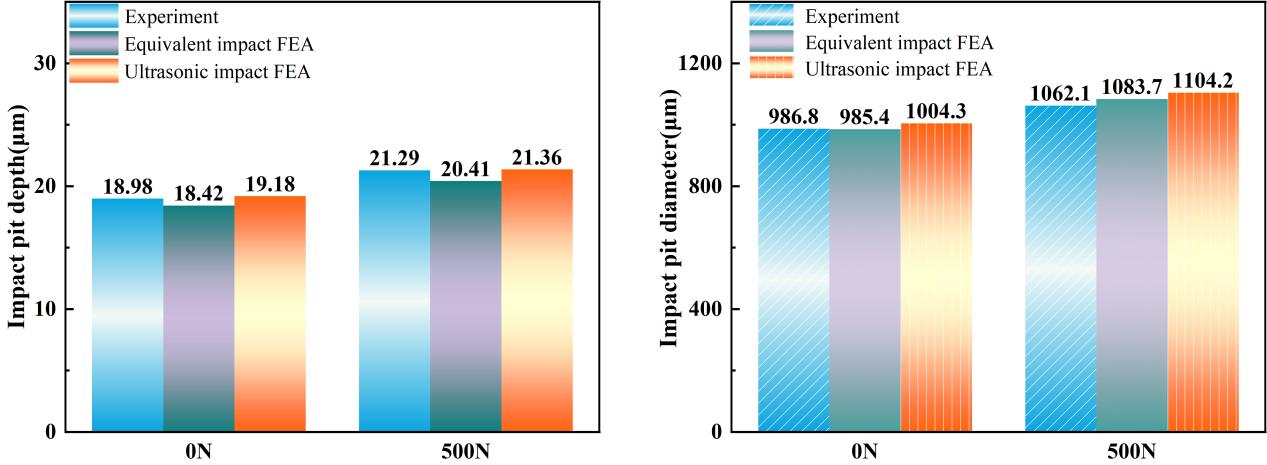


**Fig. 7.** Summary of FEA and experimental results for 45# steel.
